# Real-world treatment patterns for patients with non-infectious uveitis in Japan: a descriptive study using a large-scale claims database (J-CAT study)

**DOI:** 10.1186/s12348-025-00514-5

**Published:** 2025-07-22

**Authors:** Sentaro Kusuhara, Koh-Hei Sonoda, Toshikatsu Kaburaki, Tachie Fujita, Saki Katayama, Misako Makishima, Takao Nakamura, Mariko Nio, Takashi Omoto, Yukari Matsuo-Tezuka, Tomoki Yoshizaki, Kensuke Sasaki, Kairi Ri, Keiko Sato, Hiroshi Goto

**Affiliations:** 1 Division of Ophthalmology, Department of Surgery, Kobe University Graduate School of Medicine, 7-5-1 Kusunoki-Cho, Chuo-Ku, Kobe, 650-0017 Japan; 2 Department of Ophthalmology, Graduate School of Medical Science, Kyushu University, Fukuoka, Japan; 3 Jichi Medical University, Tochigi, Japan; 4 Chugai Pharmaceutical Co., Ltd., Tokyo, Japan; 5 Datack, Inc., Tokyo, Japan; 6 Third Place, LLC, Shizuoka, Japan; 7 Department of Ophthalmology, Tokyo Medical University, Tokyo, Japan

**Keywords:** Corticosteroid, Glaucoma, Japan, Non-infectious uveitis, Treatment patterns

## Abstract

**Purpose:**

Non-infectious uveitis (NIU) can arise from various inflammatory disorders and can cause vision loss. Patients with mild NIU are typically treated with corticosteroid eye drops to reduce intraocular inflammation; however, other local/systemic treatments (corticosteroids, immunosuppressants, biologics) may be required for moderate-to-severe NIU, which may cause ocular complications. Here, we investigated real-world treatment patterns for NIU in Japan.

**Methods:**

Patients were selected from a large, Japanese insurance claims database using *International Classification of Diseases*,* Tenth Revision* codes; diagnosis of NIU was confirmed via ophthalmological examination (October 2016–October 2023). Sankey diagrams were used to describe treatment transitions. Post-treatment ocular complications, potentially related surgeries, and fundus findings associated with uveitis were determined.

**Results:**

The majority of patients (68.7%; 37,869/55,091) were treated with corticosteroid eye drops only for mild NIU; 19.0% (10,449/55,091) were given other treatments for moderate-to-severe NIU, mostly oral corticosteroids (7,473/10,449) and posterior sub-Tenon’s corticosteroid injections (1,636/10,449). In patients treated with corticosteroids orally or via sub-Tenon’s injections, common transitions were to corticosteroid eye drops or censor (end of treatment/dataset or insurance withdrawal). A higher incidence of treatment-related ocular complications and potentially related surgeries (including glaucoma) was observed during the first year of NIU treatment compared with subsequent years (for moderate-to-severe NIU, estimated incidence of prescription of glaucoma drugs was 106 per 1,000 person-years [at 1 year], 73 per 1,000 person-years [at 2 years], and 52 per 1,000 person-years [at 5 years]).

**Conclusion:**

Our comprehensive analysis of a large claims database included all prescribed medications and medical procedures (including local injections) for NIU treatment in Japan up to October 2023. Although corticosteroids are a mainstay of NIU treatment in Japan, we found that a number of treatments for moderate-to-severe NIU, other than corticosteroid eye drops, are frequently used in combination with or when switching from corticosteroid eye drops. These findings are of importance when assessing the treatment landscape and may help identify unmet clinical needs in patients with NIU.

**Supplementary Information:**

The online version contains supplementary material available at 10.1186/s12348-025-00514-5.

## Introduction

Non-infectious uveitis (NIU) arises in several inflammatory diseases, including sarcoidosis, Vogt-Koyanagi-Harada (VKH) disease, Behçet’s disease, and systemic immune disorders [1, 2], and may cause vision loss in patients [3, 4]. Despite the widespread availability of NIU treatments worldwide, the current clinical and economic burden of this disease is high [5], and approved treatment options available for patients with NIU in many countries, including Japan, are limited [6].

Most cases of NIU are treated only with corticosteroid eye drops to reduce intraocular inflammation [6]; however, other treatments such as local and systemic administration of corticosteroids, immunosuppressants, and biologics may also be required [7]. Local and systemic corticosteroids are the most common treatments for moderate-to-severe cases of NIU, with local delivery often being used to treat posterior uveitis (for example, sub-Tenon’s injections of triamcinolone acetonide [STTA], which are often used to treat uveitic macular edema [UME], as well as corticosteroid implants) [6, 8] and systemic delivery being used to treat both NIU and associated systemic disorders. Other treatments that may be given to patients with NIU in Japan include immunosuppressants (for example, cyclosporin A [7, 9]), tumor necrosis factor-alpha (TNF-α) inhibitors [10–12], and off-label Janus kinase (JAK) inhibitors [12, 13]. Although corticosteroids are a mainstay of NIU treatment in Japan, there is a considerable risk of ocular and systemic side effects [14, 15]. For example, in a Japanese longitudinal study of patients with sarcoidosis, 32.0% of patients required cataract surgery, 9.2% had a vitrectomy (to clear vitreous opacities), and 8.5% had glaucoma surgery over a period of 20 years, and these surgeries may have been associated with corticosteroid treatment to manage intraocular inflammation [16]. In addition, adverse events associated with systemic delivery of immunosuppressants and biologics, including infections and intraocular inflammation related to anti-drug antibodies, are also possible [17–21].

Previous real-world studies of treatment patterns for Japanese patients with NIU, based on the evaluation of data from a large, nationwide, insurance claims database (Japan Medical Data Center [JMDC]) up to May 2017, showed that most patients were prescribed corticosteroid eye drops over the first 6 months after entry into the JMDC database [22]. In addition, oral corticosteroids were primarily prescribed in Japan for short-term use (< 1 month); however, some patients continued to receive oral corticosteroids for ≥ 1 year [23]. Although these previous studies reported the use of prescribed topical or oral corticosteroids, the use of local corticosteroid injections, which are an important part of NIU treatment in Japan, as well as use of immunosuppressants and biologics in clinical practice is not understood. For example, treatment patterns of adalimumab (a TNF-α inhibitor) in Japan remain unclear because it was not approved at the time of existing real-world studies. Furthermore, the risk and frequency of side effects, including ocular complications and potentially related surgeries, after administration of NIU treatments in Japan remains unclear.

Understanding treatment patterns and associated ocular complications could help recognize unmet clinical needs in patients with NIU, especially those with moderate-to-severe disease. Here, we aim to understand recent treatment patterns for NIU in a real-world setting in Japan, using data from the JMDC database, one of the largest insurance claims databases in Japan.

## Methods

### Study design and data source

This longitudinal, descriptive study (Japanese Medical Database Study to Clarify Actual Treatment Patterns in Patients with Non-Infectious Uveitis: J-CAT study, #jRCT1030240195) utilized health insurance claims between January 1, 2005 and October 31, 2023, provided by JMDC Inc. (Tokyo, Japan; data extraction in July 2024). The JMDC database is one of the largest nationwide insurance claims databases used for real-world studies in Japan [24, 25]. As of October 2023, this database included ~ 20 million employees and their dependents covered by health insurance plans. Of note, individuals aged ≥ 75 years are not included in this database because they are covered by a separate national medical insurance system.

The study was exempt from Ethics Review Board review and approval because anonymized data were used.

### Study population

Patients were included in the analysis if they had: an NIU-related claim with any *International Classification of Diseases*,* Tenth Revision (ICD-10)* code specified in Table S1 (list of disorders that may cause NIU, data extraction step 1) between January 1, 2005 and October 31, 2023; an NIU-related disease name specified in Table S2 (data extraction step 2); and an initial ophthalmological examination record specified in Table S3 within the same month of diagnosis between October 1, 2016 and October 31, 2023 (index date, also referred to as the first encounter) to confirm NIU diagnosis (data extraction step 3). A look-back period of 6 months before the index date was used to identify patients with new-onset NIU. Patients were excluded if they had: *ICD-10* codes of infectious uveitis (Table S4) in any month after the index date month; did not have *ICD-10* codes of NIU in any month after the index date month; or had < 180 days (~ 6 months) of continuous enrollment in the database before the index date.

Patients with NIU who were treated with corticosteroid eye drops only were classified as having “mild” disease in this study. Patients prescribed any of the following treatments (including those used off-label for NIU in Japan) were classified as having “moderate-to-severe” disease in this study: local corticosteroids (posterior sub-Tenon’s, subconjunctival, intravitreal, or other local injection; note that prescription records were combined with procedure records for local injections); systemic corticosteroids (oral administration or intravenous injection); immunosuppressants (oral or eye drops); phosphodiesterase-4 (PDE4) inhibitors; JAK inhibitors; colchicine (an alkaloid); and/or TNF-α inhibitors (infliximab and adalimumab). A list of NIU treatment-related codes is provided in Table S5. Of note, to improve the specificity of treatments for NIU, systemic corticosteroids, oral immunosuppressants, and biologics were only included after the prescription of eye drops or local corticosteroid injections for patients with moderate-to-severe NIU, except for VKH disease, posterior scleritis, and sympathetic ophthalmitis (as other systemic treatments may be used to treat these diseases).

### Analysis

Treatment patterns in patients diagnosed with NIU-related diseases were analyzed throughout the study period (first encounter [index date] to last encounter). Patients were followed from the index date until disenrollment of health insurance or end of the study period (October 31, 2023), whichever came first. Sankey diagrams were used to describe treatment transitions, including discontinuation, switch, removal, add-on, and censor (end of treatment, end of data set, or lack of treatment data due to insurance withdrawal). With the exception of Fig. 4, the Sankey diagrams do not show concomitant use of steroid eye drops.

Using claims data (specific *ICD-10* codes), we also investigated prevalence of NIU-associated UME in the JMDC database, estimated incidence of ocular complications and potentially related surgeries after treatment (including glaucoma, cataract and vitreous surgery, and infections), and fundus findings associated with uveitis (including incidence of UME and epiretinal membrane [ERM]). Patients with any treatment- or uveitis-related ocular disease or potentially related surgery prior to NIU diagnosis were excluded from the incidence analysis. When calculating the incidence estimates, patients with mild or moderate-to-severe NIU were followed from the first date of NIU treatment to the disenrollment of health insurance, end of the study period, or the occurrence of the ocular complications and potentially related surgeries. The first occurrence of ocular complications and potentially related surgeries was investigated within 6 months before the treatment initiation, 1–5 years after treatment initiation, and at the end of the follow-up period. Incidence estimates were also determined for the oral corticosteroid and sub-Tenon’s corticosteroid injection subgroups, which included patients who had a history of these treatments. The first date of oral corticosteroid and sub-Tenon’s corticosteroid injection is considered as the start date of the follow-up date. *ICD-10* codes for glaucoma, UME, and ERM are provided in Table S6, and *ICD-10* codes for infections have been described previously [26–28]. Incidence rates are presented as estimates (95% confidence intervals [CI]) per 1,000 person-years (PY). Descriptive analysis was used in this study, and no formal statistical significance testing was performed.

## Results

### Patient characteristics

**Fig. 1:**
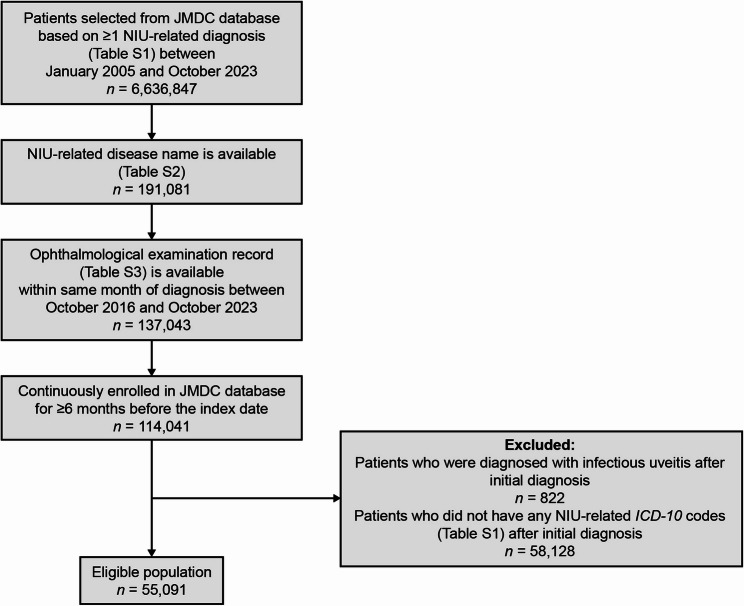
Patient attrition of eligible patients with NIU from the JMDC database. The index date, also referred to as the first encounter, is the date of the initial ophthalmological examination record (between October 1, 2016 and October 31, 2023) confirming NIU diagnosis. *ICD, International Classification of Diseases*, *Tenth Revision*; JMDC, Japan Medical Data Center; NIU, non-infectious uveitis

**Fig. 2:**
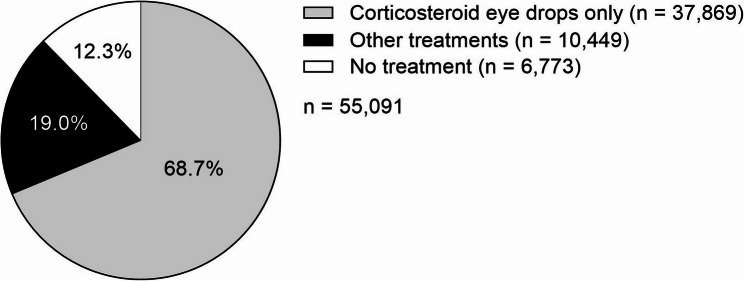
Proportion of all eligible patients with NIU in the JMDC database receiving corticosteroid eye drops (for mild NIU), other treatments (for moderate-to-severe NIU), or no treatment for NIU. JMDC, Japan Medical Data Center; NIU, non-infectious uveitis

A total of 55,091 patients with an NIU-related diagnosis met the eligibility criteria (Fig. 1). In 2023 (*N* = 13,278,370), the estimated (95% CI) prevalence of NIU in the JMDC database was 313.2 (310.2–316.3) per 100,000 persons, and estimated (95% CI) prevalence of UME was 20.9 (20.1–21.7) per 100,000 persons. For the overall cohort, the mean (± standard deviation [SD]) age was 44.9 (16.9) years, with nearly half of all patients aged 40–59 years (Table 1), and half (50.9%) were male. Most patients were given at least one treatment for NIU (87.7%, 48,318/55,091), with 68.7% of the overall cohort given treatment for mild NIU with corticosteroid eye drops only (37,869/55,091), and a further 19.0% (10,449/55,091) were given other treatments for moderate-to-severe NIU throughout the study period (Table 1; Fig. 2). The mean (± SD) time from the index date to the end of the observation period was 1,018.3 (678.3) days (or a mean 2.8 years) for the overall cohort, 999.5 (674.2) days (or a mean 2.7 years) for patients with mild NIU, and 1,168.5 (688.9) days (or a mean 3.2 years) for patients with moderate-to-severe NIU (Table 1).

**Table 1:** Patient demographics and clinical characteristics (at index date^a^) of patients with NIU

Characteristic, *n* (%)^a^	Overall *n* = 55,091	Mild NIU *n* = 37,869	Moderate-to-severe NIU *n* = 10,449
Age, mean (± SD)	44.9 (± 16.90)	44.6 (± 17.10)	46.2 (± 15.10)
Age group			
0–19 y	6,481 (11.76)	4,731 (12.49)	774 (7.41)
20–39 y	11,037 (20.00)	7,576 (20.00)	2,189 (20.95)
40–59 y	26,678 (48.40)	18,051 (47.70)	5,522 (52.80)
≥ 60 y	10,895 (19.80)	7,511 (19.80)	1,964 (18.80)
Sex, male	28,016 (50.90)	19,373 (51.20)	5,153 (49.30)
NIU diagnosis^b^			
Sarcoidosis	1,754 (3.18)	515 (1.36)	342 (3.27)
Behçet’s disease	1,099 (2.00)	134 (0.35)	363 (3.47)
Posner-Schlossman syndrome	754 (1.37)	619 (1.64)	81 (0.78)
Vogt-Koyanagi-Harada disease	639 (1.16)	60 (0.16)	534 (5.11)
Acute anterior uveitis	316 (0.57)	192 (0.51)	115 (1.10)
Diabetic iritis	63 (0.11)	35 (0.09)	13 (0.12)
Multiple Evanescent White Dot Syndrome	17 (0.03)	3 (0.01)	4 (0.04)
Rheumatoid arthritis-associated uveitis	11 (0.02)	4 (0.01)	5 (0.05)
Fuchs iridocyclitis	7 (0.01)	7 (0.02)	0
Other^c^			
Scleritis/episcleritis	20,061 (36.40)	15,934 (42.10)	3,319 (31.76)
Unclassified uveitis	15,149 (27.50)	9,195 (24.28)	4,075 (39.00)
Iritis	11,206 (20.34)	7,997 (21.12)	1,794 (17.17)
Iridocyclitis	6,042 (10.97)	4,430 (11.70)	925 (8.85)
Chorioretinitis	418 (0.76)	227 (0.60)	50 (0.48)
Diagnosis with systemic disorder associated with NIU^d, e^	4,767 (8.65)	2,382 (6.29)	1,226 (11.73)
Diabetes mellitus, unspecified type	1,866 (3.39)	1,171 (3.09)	430 (4.12)
Spondylosis	1,353 (2.46)	848 (2.24)	339 (3.24)
Sarcoidosis	563 (1.02)	120 (0.32)	83 (0.79)
Treatment for systemic disorders related to NIU^d^	3,611 (6.55)	766 (2.02)	1,924 (18.41)
Uveitic macular edema^d^	480 (0.87)	229 (0.61)	154 (1.47)
Epiretinal membrane^d^	1,018 (1.85)	669 (1.77)	200 (1.91)
Time (days) from index date to end of observation period, mean (± SD)	1,018.3 (678.33)	999.5 (674.20)	1,168.5 (688.94)

*NIU* non-infectious uveitis, *SD* standard deviation

^a^Unless stated

^b^Some patients were diagnosed with multiple conditions

^c^Top five diagnoses under “other” are listed

^d^≤ 6 months before index date

^e^Systemic disorders presenting in ≥ 1% of patients are listed

Of patients with NIU diagnoses most commonly reported in Japan [29], 3.2% were confirmed to have sarcoidosis, 2.0% Behçet’s disease, and 1.2% VKH disease. Approximately 95% of patients had “other” NIU diagnoses, including scleritis/episcleritis, unclassified uveitis, iritis, and iridocyclitis. For uveitis-related ocular diseases, < 1% of patients had UME and < 2% had ERM in the 6 months before the study period. Overall, 8.7% of patients (4,767/55,091) were diagnosed with a systemic disorder associated with NIU (*ICD-10* codes listed in Table S7) during the 6 months before NIU diagnosis, most commonly diabetes and spondylosis. However, for moderate-to-severe NIU, a larger proportion of patients were diagnosed with a systemic disorder (11.7%) than patients with mild NIU (6.3%). In addition, 18.4% of patients with moderate-to-severe NIU had a history of treatment for systemic disorders related to NIU (defined in Table S7), versus 2.0% of patients with mild NIU.

### Treatment selection

**Fig. 3:**
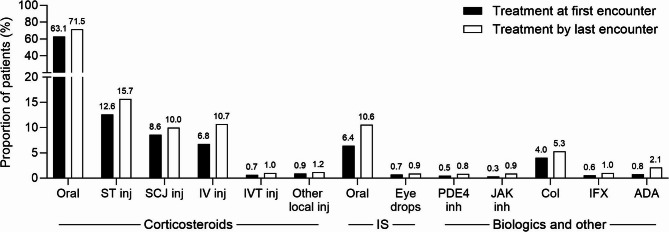
Proportion of patients with moderate-to-severe NIU receiving treatments other than corticosteroid eye drops. Treatments taken at the first encounter (index date) and all treatments taken by the last encounter are both presented (note that patients may have received multiple treatments). Infliximab and adalimumab are TNF-α inhibitors. ADA, adalimumab; Col, colchicine; IFX, infliximab; inh, inhibitor; inj, injection; IS, immunosuppressant; IV, intravenous; IVT, intravitreal; JAK, Janus kinase; NIU, non-infectious uveitis; PDE4, phosphodiesterase-4; SCJ, subconjunctival; ST, sub-Tenon’s; TNF-α, tumor necrosis factor-alpha

Among patients with moderate-to-severe NIU, the most common initial treatments (except for corticosteroid eye drops) were oral corticosteroids (63.1%), followed by local corticosteroid injections (posterior sub-Tenon’s [12.6%] and subconjunctival [8.6%]), intravenous injections (6.8%), oral immunosuppressants (6.4%), and colchicine (4.0%) (Fig. 3, Tables S8 and S9). Patients with UME or ERM in the 6 months before NIU diagnosis were more likely to receive initial treatment with posterior sub-Tenon’s corticosteroid injections (UME: 49.4%, 76/154; ERM: 36.5%, 73/200) than all patients with moderate-to-severe NIU (12.6%, 1,320/10,449). By the end of the study, the most common treatments for patients with moderate-to-severe NIU (except for corticosteroid eye drops) were generally consistent with initial treatments used: oral corticosteroids (71.5%), local corticosteroid injections (posterior sub-Tenon’s [15.7%] and subconjunctival [10.0%]), intravenous injections (10.7%), oral immunosuppressants (10.6%), and colchicine (5.3%) (Fig. 3, Tables S10 and S11).

### Treatment transitions

**Fig. 4:**
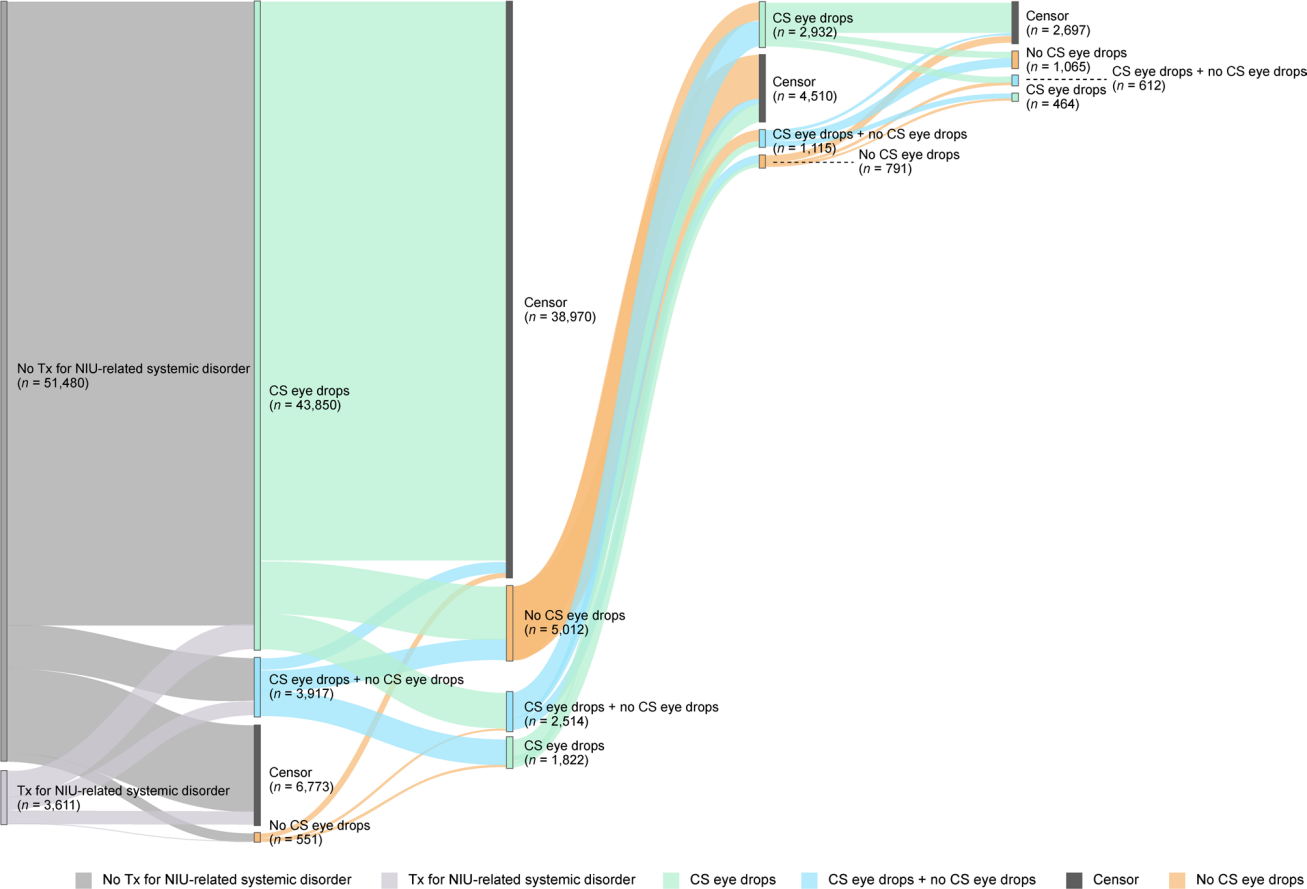
Treatment patterns of patients with moderate-to-severe NIU in the JMDC database. Sankey diagrams showing treatment transitions in the cohort that previously received corticosteroid eye drops and were subsequently given other treatments than corticosteroid eye drops. Only nodes of *n* = ≥10 are shown. CS, corticosteroid; JMDC, Japan Medical Data Center; NIU, non-infectious uveitis; Tx, treatment

Of the patients who received corticosteroid eye drops as their initial treatment (line 1), 86.4% (37,869/43,850) were only treated with corticosteroid eye drops for mild NIU. The other 13.6% (5,981/43,850) subsequently switched to other treatments (line 2) (Fig. 4) and were regarded as having moderate-to-severe NIU. Some patients with moderate-to-severe NIU (42.8%, 4,468/10,449) received treatments other than corticosteroid eye drops as their initial treatment.

#### Treatment transitions: systemic corticosteroids

**Fig. 5:**
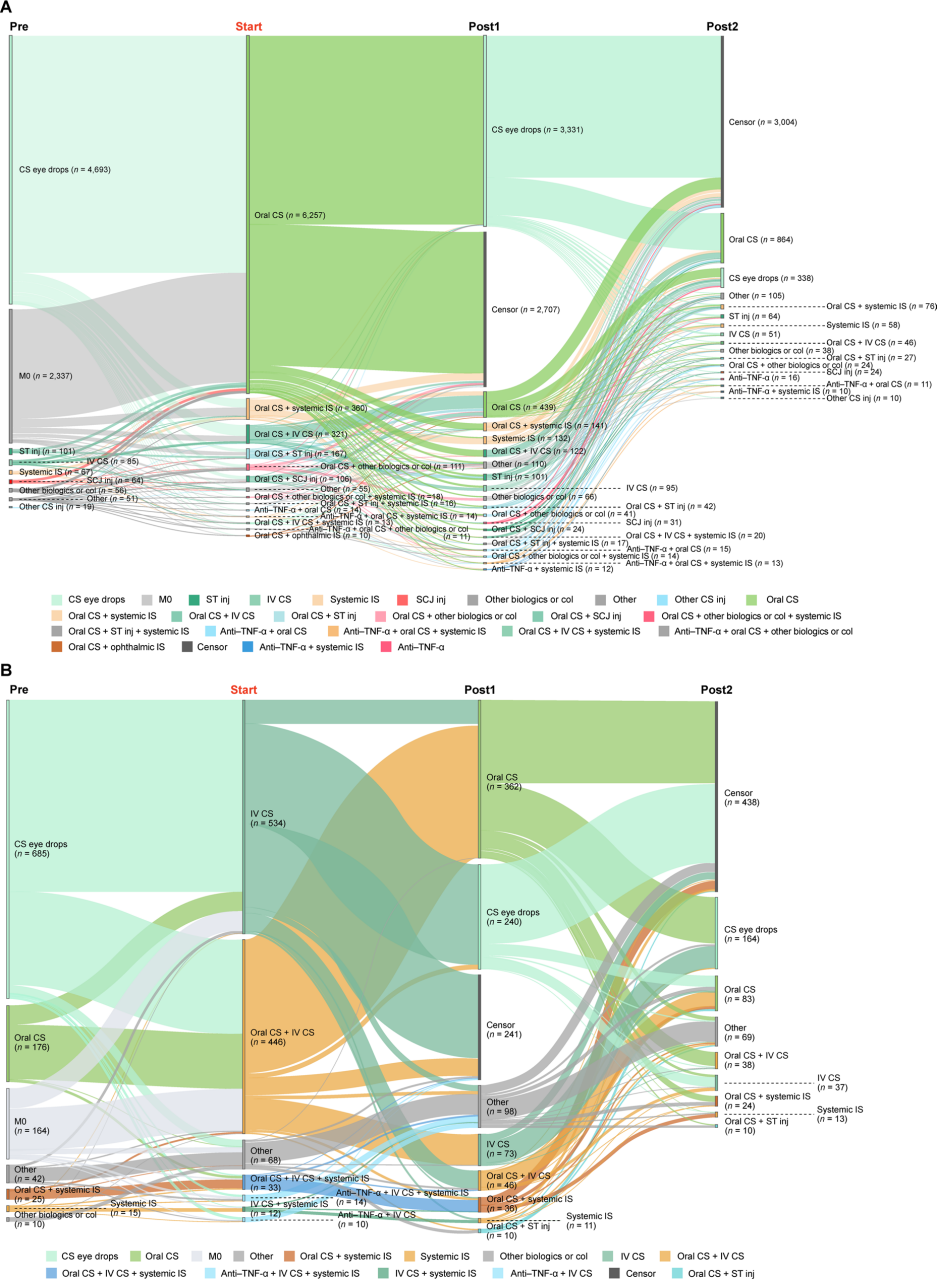
Treatment patterns in patients with moderate-to-severe NIU receiving systemic corticosteroids. Sankey diagrams showing treatment transitions before and after initiation of corticosteroids via (**A**) oral administration and (**B**) intravenous infusion. Only nodes of *n* = ≥10 are shown. Col, colchicine; CS, corticosteroid; inj, injection; IS, immunosuppressant; IV, intravenous; M0; no previous treatment; NIU, non-infectious uveitis; SCJ, subconjunctival; ST, sub-Tenon’s; TNF-α, tumor necrosis factor-alpha

For patients with moderate-to-severe NIU receiving oral corticosteroids at least once during the study period (71.5%, 7,473/10,449), 83.7% (6,257/7,473) of patients were started on oral corticosteroids alone or in combination with corticosteroid eye drops (Fig. 5A). Among these 6,257 patients, 3,331 (53.2%) subsequently transitioned to corticosteroid eye drops, and 2,707 (43.3%) were censored. The remaining 219 patients (3.5%) required other treatments (only treatments given to groups of *≥* 10 patients are shown). In patients who switched to corticosteroid eye drops alone after oral corticosteroids (44.2%, 3,303/7,473), 74.7%, 12.2%, and 13.1% were treated with oral corticosteroids for < 1 month, ≥ 1 month to < 3 months, or ≥ 3 months, respectively, before switching. In patients who switched to censor after oral corticosteroids (33.1%, 2,477/7,473), 45.2%, 12.1%, and 42.7% of patients were treated with oral corticosteroids for < 1 month, ≥ 1 month to < 3 months, or ≥ 3 months, respectively, before switching. During the first year of treatment, 13.0% (1,358/10,449) of patients with moderate-to-severe NIU were on high-dose oral corticosteroids (≥ 7.5 mg/day) (Table 2), which decreased to 2.6% (193/7,441) during the second year of treatment.

**Table 2:** Steroid treatment burden in patients with moderate-to-severe NIU

*n* (%)	1 year of treatment^a^ *n* = 10,449
High-dose oral corticosteroids (≥ 7.5 mg/day)	1,358 (13.00)
Number of corticosteroid sub-Tenon’s injections	
None	8,874 (84.93)
1	1,023 (9.79)
2	353 (3.38)
≥ 3	199 (1.90)

*NIU* non-infectious uveitis

^a^During the first year of treatment after the index date

For patients receiving a corticosteroid drip infusion (intravenous injection, 10.7%, 1,117/10,449), patients were mostly given intravenous corticosteroids either alone or in combination with oral corticosteroids (87.7%, 980/1,117) (Fig. 5B). Although many of these patients switched from corticosteroid eye drops to intravenous corticosteroids, the majority of patients subsequently switched away from intravenous corticosteroids to either oral corticosteroids or corticosteroid eye drops (61.4%, 602/980).

#### Treatment transitions: local corticosteroid injections

**Fig. 6:**
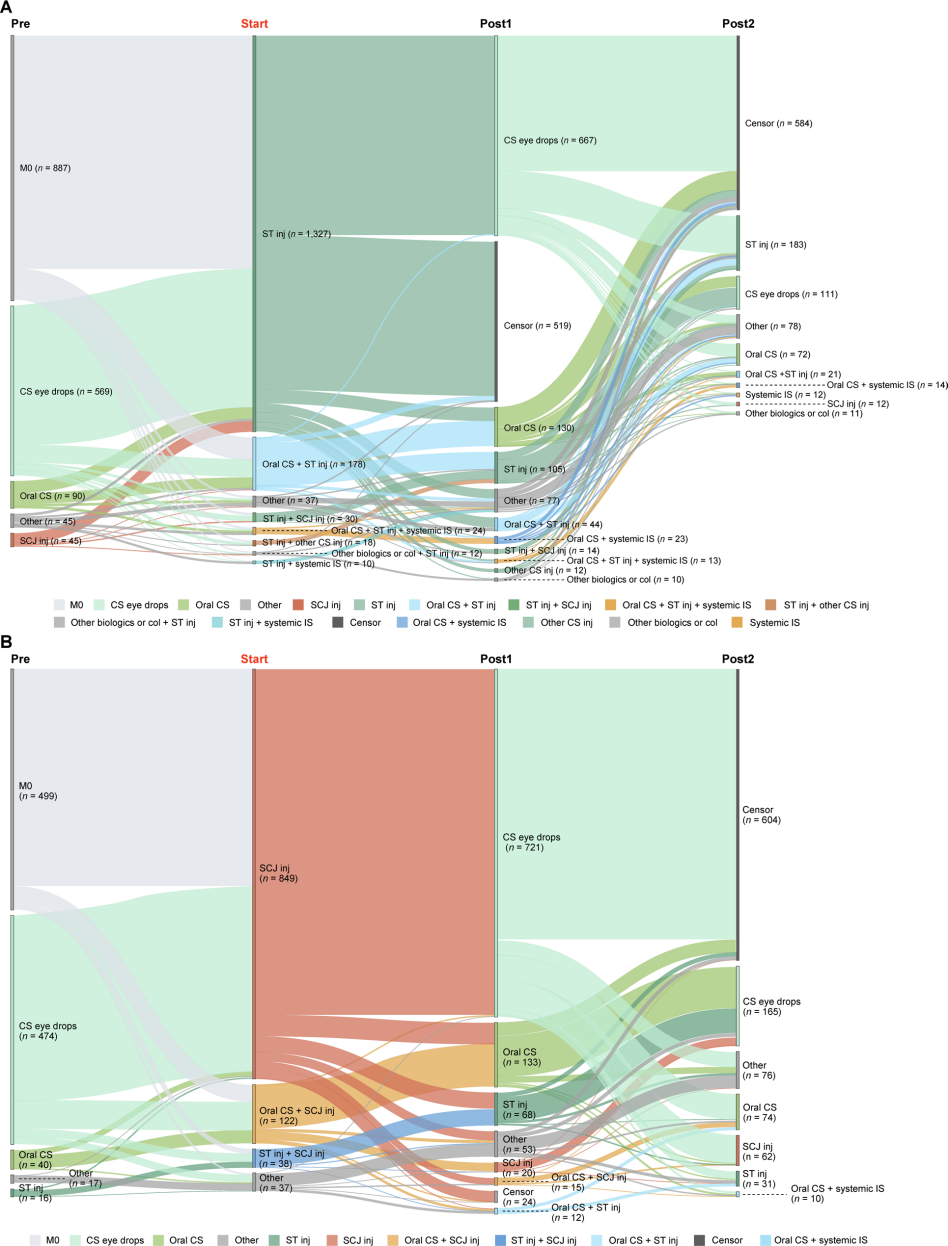
Treatment patterns in patients with moderate-to-severe NIU receiving local corticosteroid injections. Sankey diagrams showing treatment transitions before and after initiation of corticosteroids via (**A**) sub-Tenon’s injections and (**B**) subconjunctival injections. Only nodes of *n* = ≥10 are shown. Col, colchicine; CS, corticosteroid; inj, injection; IS, immunosuppressant; M0; no previous treatment; NIU, non-infectious uveitis; SCJ, subconjunctival; ST, sub-Tenon’s

For patients with moderate-to-severe NIU who received posterior corticosteroid sub-Tenon’s injections at least once during the study period (15.7%, 1,636/10,449), patients were most commonly treated with corticosteroid eye drops before and after the sub-Tenon’s injections (Fig. 6A). In patients who switched to corticosteroid eye drops after sub-Tenon’s injections (40.8%, 667/1,636), 69.9%, 19.9%, and 10.2% received one, two, and three or more injections, respectively, before switching. In patients who switched to censor after sub-Tenon’s injections (31.7%, 519/1,636), 66.9%, 19.5%, and 13.7% received one, two, and three or more injections, respectively, before switching.

For patients with moderate-to-severe NIU receiving corticosteroid subconjunctival injections at least once during the study period (10.0%, 1,046/10,449), patients were most commonly treated with corticosteroid eye drops before and after the subconjunctival injections (Fig. 6B). In patients who switched to corticosteroid eye drops after subconjunctival injections (68.5%, 717/1,046), 66.1%, 20.4%, and 13.5% received one, two, and three or more injections, respectively, before switching.

#### Treatment transitions: TNF-α inhibitors

**Fig. 7:**
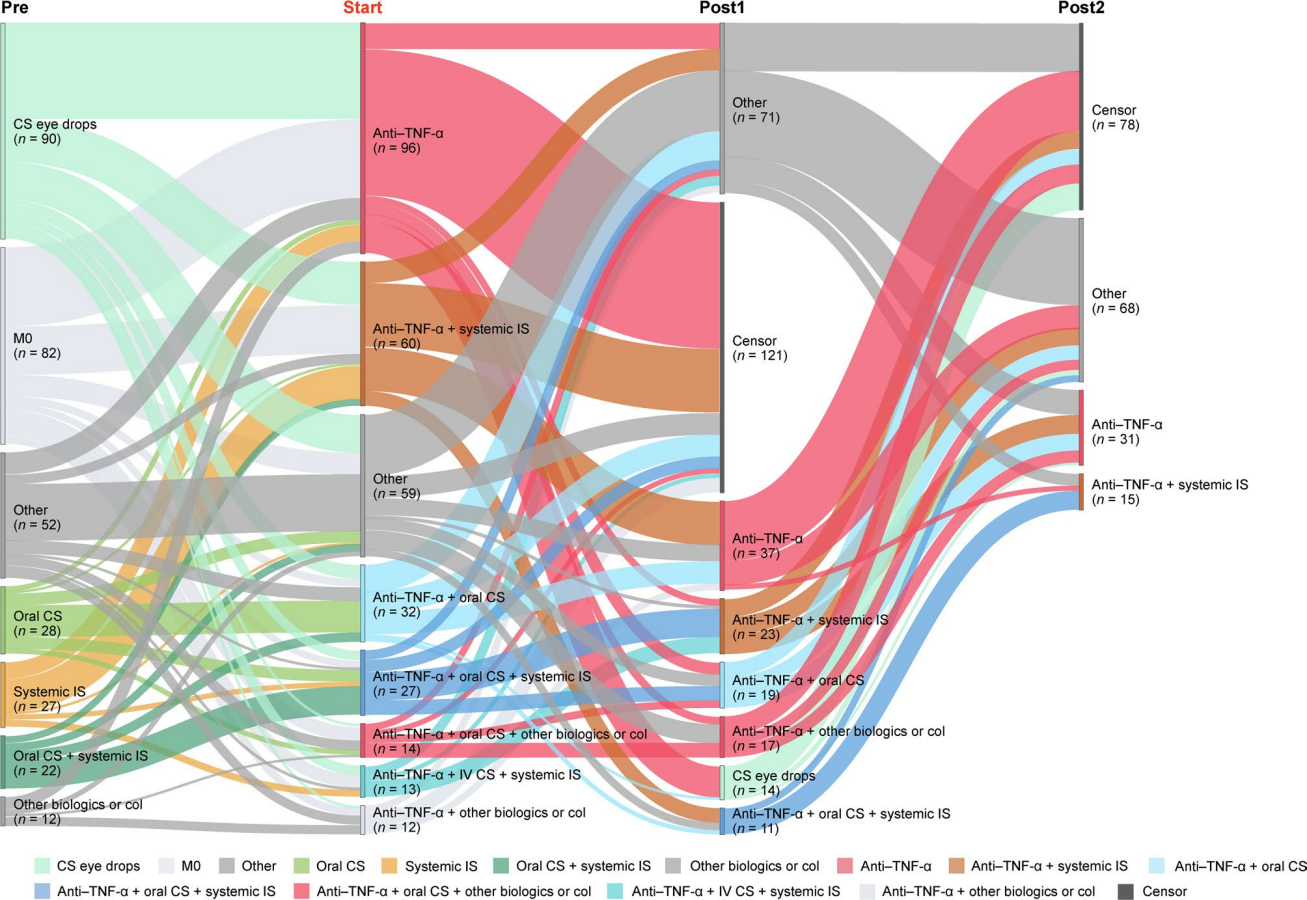
Treatment patterns in patients with moderate-to-severe NIU receiving TNF-α inhibitors (adalimumab or infliximab). Sankey diagram shows treatment transitions before and after initiation of TNF-α inhibitors. Only nodes of *n* = ≥10 are shown. Col, colchicine; CS, corticosteroid; IS, immunosuppressant; IV, intravenous; M0; no previous treatment; NIU, non-infectious uveitis; TNF-α, tumor necrosis factor-alpha

For patients with moderate-to-severe NIU who were treated with TNF-α inhibitors at least once during the study period (313/10,449, adalimumab *n* = 220 or infliximab *n* = 108, some patients received both TNF-α inhibitors during the study period), 69.3% (217/313) of patients received concomitant medications, including systemic immunosuppressants, oral corticosteroids, and/or other treatments (Fig. 7 and Fig. S1). TNF-α inhibitors were the initial treatment in 26.2% (82/313) of patients who received TNF-α inhibitors during the study period. In patients who switched to censor after treatment with TNF-α inhibitors, 70.5% (43/61) were treated for ≥ 12 months.

### Treatment lines for NIU

**Fig. 8:**
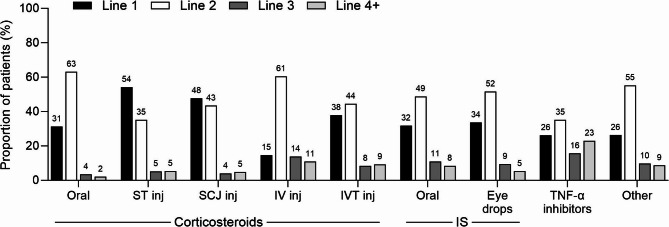
Proportion of patients with moderate-to-severe NIU receiving treatments other than corticosteroid eye drops at each line. Inj, injection; IS, immunosuppressant; IV, intravenous; IVT, intravitreal; NIU, non-infectious uveitis; SCJ, subconjunctival; ST, sub-Tenon’s; TNF-α, tumor necrosis factor-alpha

We also investigated treatment lines for patients with NIU (for all treatments except corticosteroid eye drops). Posterior sub-Tenon’s and subconjunctival corticosteroid injections were most common at treatment line 1 for patients with moderate-to-severe NIU (Fig. 8), whereas systemic (oral/intravenous) corticosteroids, intravitreal corticosteroids, and immunosuppressants were most common at treatment line 2 (Fig. 8).

### Incidence of ocular diseases associated with NIU or its treatment

The overall estimated incidence rate of treatment-related ocular diseases and potentially related surgeries was higher during the first year of NIU treatment (Table 3) than years 2–5 of treatment. Prescription of glaucoma drugs was common in patients with mild and moderate-to-severe NIU and was highest during the first year of treatment (estimated incidence was 106–119 per 1,000 PY at 1 year, 73–74 per 1,000 PY at 2 years, and 50–52 per 1,000 PY at 5 years). During the first year of NIU treatment, cataract surgery was more common in patients with mild NIU (52 per 1,000 PY) than in patients with moderate-to-severe NIU (31 per 1,000 PY). The incidence of prescription of glaucoma drugs, glaucoma surgery, cataract surgery, and vitreous surgery during years 1–5 of treatment was higher in patients who received posterior sub-Tenon's corticosteroid injections than in all patients with moderate-to-severe NIU. For example, estimated incidence of prescription of glaucoma drugs in patients receiving sub-Tenon's corticosteroid injections was 235 per 1,000 PY at 1 year, 166 per 1,000 PY at 2 years, and 117 per 1,000 PY at 5 years.

**Table 3:** Estimated (95% CI) incidence rate^a^ of treatment-related ocular diseases and potentially related surgeries in patients with NIU

	Mild NIU *n *= 37,869			Moderate-to-severe NIU *n* = 10,449		
	1 year of treatment^b^	2 years of treatment^b^	5 years of treatment^b^	1 year of treatment^b^	2 years of treatment^b^	5 years of treatment^b^
Prescription of glaucoma drugs	119.4 (115.5–123.3)	74.2 (71.9–76.5)	49.6 (48.1–51.1)	105.6 (98.7–112.8)	73.2 (68.7–77.8)	52.3 (49.3–55.5)
Glaucoma surgery	11.9 (10.8–13.1)	7.8 (7.1–8.5)	5.5 (5.0–6.0)	10.1 (8.1–12.4)	8.1 (6.8–9.7)	5.9 (4.9–6.9)
Cataract surgery	51.9 (49.5–54.5)	33.4 (31.9–35.0)	23.7 (22.7–24.7)	30.8 (27.3–34.7)	29.0 (26.3–31.9)	23.9 (22.0–26.0)
Vitreous surgery	14.8 (13.5–16.2)	9.4 (8.7–10.3)	6.5 (6.0–7.0)	15.9 (13.4–18.7)	12.4 (10.7–14.4)	9.2 (8.0–10.5)
Infections	146.8 (142.6–151.1)	126.7 (123.7–129.8)	110.8 (108.5–113.2)	239.0 (228.3–250.1)	198.0 (190.3–205.9)	167.8 (161.9–173.9)

	Oral corticosteroids *n* = 6,588			Steroids for sub-Tenon's injection *n* = 1,320		
	1 year of treatment^b^	2 years of treatment^b^	5 years of treatment^b^	1 year of treatment^b^	2 years of treatment^b^	5 years of treatment^b^
Prescription of glaucoma drugs	87.1 (79.7–94.9)	61.20 (56.4–66.3)	44.50 (41.2–48.0)	235.4 (209.1–264.2)	165.6 (148.3–184.4)	117.3 (105.3–130.3)
Glaucoma surgery	8.8 (6.6–11.4)	6.6 (5.2–8.4)	4.8 (3.8–6.0)	17.8 (11.5–26.3)	17.7 (12.7–23.9)	12.5 (9.1–16.7)
Cataract surgery	20.9 (17.5–24.8)	20.8 (18.1–23.7)	18.4 (16.4–20.6)	81.4 (67.0–98.1)	81.3 (69.9–93.9)	65.7 (57.3–74.9)
Vitreous surgery	7.7 (5.7–10.2)	6.7 (5.3–8.5)	5.1 (4.1–6.3)	61.1 (48.7–75.7)	44.7 (36.5–54.3)	34.5 (28.7–41.3)
Infections	262.0 (248.7–276.1)	216.0 (206.3–225.9)	184.0 (176.8–192.0)	165.9 (144.5–189.5)	142.7 (127.2–159.7)	122.1 (110.1–135.1)

Patients with any treatment-related ocular disease or potentially related surgery prior to NIU diagnosis were excluded

*CI* confidence interval, *NIU* non-infectious uveitis

^a^Per 1,000 person-years

^b^From the index date

### Uveitis-related ocular diseases

The estimated incidence rate of UME was higher in patients with moderate-to-severe NIU (69 per 1,000 PY) than in patients with mild NIU (32 per 1,000 PY), particularly during the first year of treatment (Table 4). The estimated incidence of UME during years 1–5 of NIU treatment was also higher in patients who received posterior sub-Tenon's corticosteroid injections (260 per 1,000 PY at 1 year, 166 per 1,000 PY at 2 years, and 112 per 1,000 PY at 5 years) than in all patients with moderate-to-severe NIU (69 per 1,000 PY at 1 year, 46 per 1,000 PY at 2 years, and 32 per 1,000 PY at 5 years). The incidence of ERM generally remained unchanged during years 1–5 of NIU treatment and was higher in patients who received posterior sub-Tenon's corticosteroid injections (46 per 1,000 PY at 1 year) than in all patients with moderate-to-severe NIU (19 per 1,000 PY at 1 year).

**Table 4:** Estimated (95% CI) incidence rate^a^ of uveitis-related ocular diseases in patients with NIU

	Mild NIU *n* = 37,869			Moderate-to-severe NIU *n* = 10,449		
	1 year of treatment^b^	2 years of treatment^b^	5 years of treatment^b^	1 year of treatment^b^	2 years of treatment^b^	5 years of treatment^b^
Uveitic macular edema	31.6 (29.7–33.6)	19.9 (18.7–21.1)	13.6 (12.8–14.3)	68.8 (63.3–74.6)	45.7 (42.2–49.3)	31.8 (29.5–34.3)
Epiretinal membrane	22.9 (21.3–24.6)	16.9 (15.9–18.0)	13.2 (12.5–14.0)	19.3 (16.5–22.4)	16.6 (14.6–18.8)	14.2 (12.7–15.8)

	Oral corticosteroids *n* = 6,588			Steroids for sub-Tenon's injection *n* = 1,320		
	1 year of treatment^b^	2 years of treatment^b^	5 years of treatment^b^	1 year of treatment^b^	2 years of treatment^b^	5 years of treatment^b^
Uveitic macular edema	36.5 (31.8–41.6)	25.0 (22.0–28.2)	18.4 (16.3–20.6)	259.5 (231.3–290.2)	165.6 (148.2–184.4)	111.8 (100.2–124.3)
Epiretinal membrane	14.1 (11.3–17.4)	12.3 (10.2–14.6)	11.1 (9.5–12.8)	46.3 (35.7–59.1)	37.0 (29.6–45.7)	30.1 (24.7–36.4)

Patients with any uveitis-related ocular disease prior to NIU diagnosis were excluded

*CI* confidence interval, *NIU* non-infectious uveitis

^a^Per 1,000 person-years

^b^From the index date

## Discussion

To our knowledge, this is the first study to examine a wide range of recent real-world treatment patterns for patients with moderate-to-severe NIU in Japan, including local corticosteroid injections, immunosuppressants, and biologics. Using insurance claims data from patients with NIU, we found that the majority of patients were treated with corticosteroid eye drops alone (mild NIU): however, nearly 20% switched to other treatment options, including oral steroids, posterior sub-Tenon's steroid injections, immunosuppressants, and biologics/other treatments (moderate-to-severe NIU). Many of these patients may have required more effective treatments than corticosteroid eye drops, which could increase the treatment burden of NIU in clinical practice (for example, the need for local injections). Patients may have also switched to non-steroid therapies to reduce the risk of inducing ocular complications. In addition, patients with moderate-to-severe NIU who received local corticosteroid injections commonly transitioned to corticosteroid eye drops or censor, indicating treatment effectiveness; however, some patients continued to receive high/frequent doses of corticosteroids, or switched to other treatments for moderate-to-severe NIU, which may indicate persistent inflammation. Given that prescription of glaucoma drugs was more common during the first year of treatment for patients with NIU, it is possible that some treatments may contribute to ocular diseases. Although corticosteroids are a mainstay of NIU treatment in Japan, our identification of treatment patterns with a high corticosteroid burden or non-steroid treatment suggests that development of updated clinical guidelines for current and emerging NIU management in Japan may improve patient outcomes.

Our findings align with previous studies on oral corticosteroids in Japan [23]. We show that oral corticosteroids were most commonly used as a treatment option for NIU, and high doses were most common during the first year of treatment, which are largely in line with previous findings by Umazume and colleagues using the JMDC database up to May 2017 [23]. In addition, treatment with oral corticosteroids before switching to corticosteroid eye drops alone was completed in a relatively short period of time (< 1 month for 75% of patients). Longer-term maintenance therapy (≥ 3 months) with oral corticosteroids used by 13% of patients in clinical practice, which was expected and reflects a goal of minimizing adverse effects that may occur at all (and especially high) doses [8, 14, 15, 30, 31]. However, some patients who were still taking oral corticosteroids by the end of the study period had been treated for ≥ 3 months, which may have increased the risk of adverse effects in these patients.

We also describe treatment patterns of local corticosteroid injections, immunosuppressants, biologics, and other treatments for moderate-to-severe NIU using (i) recent JMDC data (up to October 2023), for which treatment patterns have not been described in existing studies, and (ii) more specific disease definitions (*ICD-10* codes) for inclusion/exclusion compared with existing studies [22]. A United States TriNetX database study reported real-world use (2013–2023) of corticosteroids, immunosuppressants, and biologics in patients with uveitis, including several off-label biologics not currently approved for use in Japan. The findings from this study generally align with our findings, with oral corticosteroids (prednisone) mostly commonly prescribed. However, stepwise treatment transitions for these patients were not reported [32]. We found that local corticosteroid injections (such as posterior sub-Tenon’s) were used in the Japanese patient cohort, especially for patients with UME. These injections may allow for a targeted posterior eye delivery and could reduce adverse effects associated with systemic delivery of corticosteroids [33]. These findings are consistent with a previous study that found a correlation between presence of UME and use of posterior sub-Tenon’s injections (STTA) in tertiary centers in Japan [34]. Although local corticosteroids (sub-Tenon’s and subconjunctival) were most commonly given using a single injection to minimize the risk of adverse events, ~ 30% of patients received multiple local corticosteroid injections, indicating that there is still a clinical concern of adverse events. Many patients transitioned to using corticosteroid eye drops after local corticosteroid injections, indicating an adequate treatment response to the local injections; however, some patients transitioned from local corticosteroid injections to other treatments, indicating persistent inflammation. Importantly, the selection of treatment strategies for patients with NIU should consider the type and location of uveitis, severity of intraocular inflammation, diagnosis with systemic disorders, and presence of ocular diseases [34–36].

For TNF-α inhibitors, which were often used as a long-term (≥ 1 year) combination therapy for patients with moderate-to-severe NIU, we observed a wide variety of treatments being used before and after TNF-α inhibitor administration, as well as several concomitant medications. In Japan, guidelines do not clearly specify the treatment line at which TNF-α inhibitors can be used, or whether to discontinue other treatments when using TNF-α inhibitors [37]. Although TNF-α inhibitors are used in patients who have an inadequate response to conventional therapies [38], we found a number of patients using TNF-α inhibitors as initial treatment for moderate-to-severe NIU, possibly to avoid adverse effects associated with other systemic treatments, including corticosteroids and immunosuppressants. In addition, difficulty in discontinuing TNF-α inhibitors after initiation could be a contributing factor to longer treatment periods [39]; however, with extended TNF-α inhibitor use, there are risks of adverse effects including development of autoimmune diseases including systemic lupus erythematosus [40] and multiple sclerosis [41], as well as paradoxical sarcoidosis [42]. Overall, updated clinical guidelines are warranted for the appropriate use of biologics for NIU in Japan.

We also describe the incidence of treatment-related ocular diseases and potentially related surgeries (including glaucoma, cataract, and vitrectomy), which were generally higher during the first year of treatment. Our findings indicate that glaucoma may be more related to treatments for NIU (e.g., corticosteroid use); however, further studies are warranted to evaluate whether a causal relationship exists. It is also noted that pars plana vitrectomy is being used increasingly for management of uveitis in clinical practice [43, 44]. Although treatment options are needed that can manage inflammation while reducing risk of side effects, high-dose treatments may still be required for management of severe inflammation [17], especially during the first year of treatment. Thus, some treatment-related ocular diseases and potentially related surgeries may be unavoidable in patients with moderate-to-severe NIU. The relatively high incidence of treatment-related ocular diseases among patients who received sub-Tenon’s corticosteroid injections may reflect the associated high treatment burden (i.e., the need for frequent injections) and the consequent inherently higher risk of complications. In addition, we found that the incidence of UME was higher in patients with moderate-to-severe NIU, and especially in patients receiving sub-Tenon's corticosteroid injections, which aligns with previous findings [33, 34]. We note that the incidence rates of UME may be overestimated in this study, because of the nature of the JMDC claims database; however, using data from JMDC in 2023, we found a similar incidence of NIU in the Japanese population compared with 2016 data from JMDC.

In a previous analysis, Umazume and colleagues [22] identified NIU patients from four segments; however, selection was restricted to patients diagnosed with NIU at least once during the year of analysis. In the “unclassifiable” category, which accounts for most of the four segments, data for patients diagnosed with sarcoidosis (*ICD-10* code, D869), uveitis (M352), or incomplete uveitis (M352) were extracted only if the claim record in the same month listed eye drops in the “uveitis drug list”. This was to distinguish these patients from patients who were prescribed systemic corticosteroids or immunosuppressive drugs for the treatment of other organ systems. In other words, many patients were selected who were diagnosed with NIU at least once during the analysis period and were prescribed eye drops in the same month. Therefore, patients who were prescribed drugs for diagnostic purposes, given drugs for other diseases, misdiagnosed, or who were inactive may have been included. On the other hand, in this J-CAT study, patients were selected if they were diagnosed based on ophthalmologic examination and claims records for target disease in at least 2 months. This reduced the possibility of including patients who were prescribed drugs for diagnostic purposes, treated for other diseases, misdiagnosed, or who were inactive, even if the insurance claims data did not include a specialist’s medical record review.

This study has several limitations, which mostly relate to the nature of health insurance claims databases and inherent issues with generalizability. We acknowledge that the estimated NIU prevalence could be an underestimation because patients who already had NIU before the assessment period (November 2016) could not be identified. Hence, the annual prevalence in the first year should be interpreted with caution, particularly for 2016–2018. The study relied on *ICD-10* codes to identify NIU, which were not validated, and laboratory tests results were not available (although patients were required to have an ocular examination within the same month of diagnosis to improve accuracy of NIU diagnosis in patients included in the study). We cannot rule out miscoding and misclassification, and the disease causing NIU was difficult to distinguish for some patients. Furthermore, we were unable to determine NIU duration, UME status, visual outcomes, and whether the disease was unilateral/bilateral, which may all affect treatment selection. Moderate-to-severe *ICD-10* codes other than VKH disease, posterior scleritis, and sympathetic ophthalmia were required to have a previous history of corticosteroid eye drops, as these diseases could be diagnosed by physicians other than ophthalmologists. In addition, the presence/absence of treatment was defined based on prescription data, but it is unknown if patients were actually administered with the treatment. Another potential limitation is the possible underrepresentation of individuals aged ≥ 75 years, as separate insurance coverage in Japan is available (though this is considered to have minimal impact on the study findings). In addition, most patients in the JMDC database were identified as having “other” NIU diagnoses such as scleritis/episcleritis, uveitis, iritis, and iridocyclitis, which may be more common in real-world data than in studies conducted in specialist facilities. Importantly, JAK inhibitors, which are included in this study as a reference drug, are not currently approved in Japan for NIU.

Despite these limitations, the JMDC database contains one of the largest cohorts of patients with NIU in Japan, providing unique insights into real-world treatment patterns for NIU. Of note, working individuals (aged 20–60 years) were well-represented in the database, which is important given they may have a higher incidence of NIU as demonstrated in our study and in previous literature [45, 46]. Furthermore, we note that the observation period (~ 3 years) was long enough to investigate disease progression to severe NIU, and to track treatment transitions over this window. Importantly, while treatment patterns identified in this study generally align with expected global clinical practice, the Sankey diagrams presented here also highlight more granular treatment patterns used by smaller groups of patients (*n* = 10–50). Some treatments were only used by a small number of patients, suggesting that treatment choices may also be based on patient-level and clinical factors. These findings show the diversity of treatment strategies used in clinical practice in Japan, which may also be applicable to global clinical practice.

In conclusion, we report real-world treatment patterns for patients with NIU in Japan using recent data (up to October 2023) from a large claims database, which could help to recognize unmet clinical needs in patients with NIU. In addition, the common use of local and systemic corticosteroids may contribute to a high treatment burden in Japan. With the current treatment landscape changing globally for patients with NIU [7], treatment efficacy and potential adverse events should be taken into consideration when assessing treatment options. Further investigation of the association between NIU treatment and ocular complications is warranted. Importantly, development of updated clinical guidelines for NIU diagnosis, management, and use of current and newer treatments in Japan may assist with improving outcomes for patients with NIU.

## Supplementary Information

Supplementary Material 1.

## Data Availability

The data were used under license for this study and are not publicly available owing to the privacy policy of JMDC Inc.

## Abbreviations

ADA: Adalimumab
ATC: Anatomical Therapeutic Chemical Classification System
Col: Colchicine
ERM: Epiretinal membrane
ICD-10: *International Classification of Diseases, Tenth Revision*
IFX: Infliximab
Inh: Inhibitor
Inj: Injection
IS: Immunosuppressant
IU: Infectious uveitis
IV: Intravenous
IVT: Intravitreal
JMDC: Japan Medical Data Center
NIU: Non-infectious uveitis
PDE4: Phosphodiesterase-4
SCJ: Subconjunctival
SD: Standard deviation
ST: Sub-Tenon’s
STTA: Sub-Tenon’s injections of triamcinolone acetonide
TNF-α: Tumor necrosis factor-alpha
UME: Uveitic macular edema
VKH: Vogt-Koyanagi-Harada
WHO: World Health Organization

## References

[1] Takeuchi M, Mizuki N, Ohno S (2021) Pathogenesis of non-infectious uveitis elucidated by recent genetic findings. Front Immunol 12:640473 33912164 10.3389/fimmu.2021.640473 PMC8072111

[2] Sonoda KH, Hasegawa E, Namba K, Okada AA, Ohguro N, Goto H (2021) Epidemiology of uveitis in japan: a 2016 retrospective nationwide survey. Jpn J Ophthalmol 65(2):184–190 33694024 10.1007/s10384-020-00809-1

[3] Hsu YR, Huang JC, Tao Y et al (2019) Noninfectious uveitis in the Asia-Pacific region. Eye (Lond) 33(1):66–77 30323327 10.1038/s41433-018-0223-z PMC6328561

[4] Niemeyer KM, Gonzales JA, Doan T, Browne EN, Rao MM, Acharya NR (2019) Time trade-off utility values in noninfectious uveitis. Am J Ophthalmol 208:47–55 31201795 10.1016/j.ajo.2019.06.005 PMC6888854

[5] Hariprasad SM, Joseph G, Gagnon-Sanschagrin P et al (2021) Healthcare costs among patients with macular oedema associated with non-infectious uveitis: a US commercial payer’s perspective. BMJ Open Ophthalmol 6(1):e000896 34786486 10.1136/bmjophth-2021-000896 PMC8587681

[6] Maruyama K (2019) Current standardized therapeutic approach for uveitis in Japan. Immunol Med 42(3):124–134 31645201 10.1080/25785826.2019.1678961

[7] Wu X, Tao M, Zhu L, Zhang T, Zhang M (2023) Pathogenesis and current therapies for non-infectious uveitis. Clin Exp Med 23(4):1089–1106 36422739 10.1007/s10238-022-00954-6 PMC10390404

[8] Brady CJ, Villanti AC, Law HA et al (2016) Corticosteroid implants for chronic non-infectious uveitis. Cochrane Database Syst Rev 2(2):CD010469 26866343 10.1002/14651858.CD010469.pub2 PMC5038923

[9] Dick AD, Rosenbaum JT, Al-Dhibi HA et al (2018) Guidance on noncorticosteroid systemic immunomodulatory therapy in noninfectious uveitis: fundamentals of care for uveitis (FOCUS) initiative. Ophthalmology 125(5):757–773 29310963 10.1016/j.ophtha.2017.11.017

[10] Cordero-Coma M, Sobrin L (2015) Anti–tumor necrosis factor-α therapy in uveitis. Surv Ophthalmol 60(6):575–589 26164735 10.1016/j.survophthal.2015.06.004

[11] Horiguchi N, Kamoi K, Horie S et al (2020) A 10-year follow-up of Infliximab monotherapy for refractory uveitis in Behçet’s syndrome. Sci Rep 10(1):22227 33335139 10.1038/s41598-020-78718-z PMC7747559

[12] Chauhan K, Tyagi M (2024) Update on non-infectious uveitis treatment: anti-TNF-alpha and beyond. Front Ophthalmol 4:1412930 10.3389/fopht.2024.1412930 PMC11327136 39157460

[13] Garweg JG, Straessle KA (2024) Janus kinase inhibitors as a third-line therapy for refractory endogenous noninfectious uveitis. Ocul Immunol Inflamm 1–8. 10.1080/09273948.2024.2348125 10.1080/09273948.2024.2348125 38709218

[14] Dick AD, Tundia N, Sorg R et al (2016) Risk of ocular complications in patients with noninfectious intermediate uveitis, posterior uveitis, or panuveitis. Ophthalmology 123(3):655–662 26712559 10.1016/j.ophtha.2015.10.028

[15] Babu K, Mahendradas P (2013) Medical management of uveitis - current trends. Indian J Ophthalmol 61(6):277–283 23803479 10.4103/0301-4738.114099 PMC3744780

[16] Takai N, Kobayashi T, Kida T, Ikeda T (2020) Clinical features of Japanese patients with ocular inflammation and their surgical procedures over the course of 20 years. Clin Ophthalmol 14:2799–2806 33061264 10.2147/OPTH.S273938 PMC7522428

[17] Agrawal H, Doan H, Pham B et al (2020) Systemic immunosuppressive therapies for uveitis in developing countries. Indian J Ophthalmol 68(9):1852–1862 32823402 10.4103/ijo.IJO_1548_20 PMC7690522

[18] Bellur S, McHarg M, Kongwattananon W, Vitale S, Sen HN, Kodati S (2023) Antidrug antibodies to tumor necrosis factor α inhibitors in patients with noninfectious uveitis. JAMA Ophthalmol 141(2):150–156 36547953 10.1001/jamaophthalmol.2022.5584 PMC9936342

[19] Nicolela Susanna F, Pavesio C (2020) A review of ocular adverse events of biological anti-TNF drugs. J Ophthalmic Inflamm Infect 10(1):11 32337619 10.1186/s12348-020-00202-6 PMC7184065

[20] Quartuccio L, Zabotti A, Del Zotto S, Zanier L, De Vita S, Valent F (2019) Risk of serious infection among patients receiving biologics for chronic inflammatory diseases: usefulness of administrative data. J Adv Res 15:87–93 30581616 10.1016/j.jare.2018.09.003 PMC6300460

[21] Penso L, Dray-Spira R, Weill A, Pina Vegas L, Zureik M, Sbidian E (2021) Association between biologics use and risk of serious infection in patients with psoriasis. JAMA Dermatol 157(9):1056–1065 34287624 10.1001/jamadermatol.2021.2599 PMC8295892

[22] Umazume A, Ohguro N, Okada AA et al (2021) Prevalence and incidence rates and treatment patterns of non-infectious uveitis in Japan: real-world data using a claims database. Jpn J Ophthalmol 65(5):657–665 34181111 10.1007/s10384-021-00850-8

[23] Umazume A, Ohguro N, Okada AA et al (2022) Use of systemic corticosteroids in patients newly registered at a claims database with a diagnosis of non-infectious uveitis: results from a real-world claims database analysis. Jpn J Ophthalmol 66(4):394–404 35670922 10.1007/s10384-022-00923-2

[24] Nagai K, Tanaka T, Kodaira N, Kimura S, Takahashi Y, Nakayama T (2021) Data resource profile: JMDC claims database sourced from health insurance societies. J Gen Fam Med 22(3):118–127 33977008 10.1002/jgf2.422 PMC8090843

[25] Laurent T, Simeone J, Kuwatsuru R, et al (2022) Context and considerations for use of two Japanese real-world databases in Japan: medical data vision and Japanese Medical Data Center. Drugs Real World Outcomes 9(2):175–187 10.1007/s40801-022-00296-5 PMC8932467 35304702

[26] Khan N, Vallarino C, Lissoos T, Darr U, Luo M (2019) Risk of infection and types of infection among elderly patients with inflammatory bowel disease: a retrospective database analysis. Inflamm Bowel Dis 26(3):462–468 10.1093/ibd/izz065 30980714

[27] Hase R, Suzuki D, de Luise C et al (2023) Validity of claims-based diagnoses for infectious diseases common among immunocompromised patients in Japan. BMC Infect Dis 23(1):653 37789253 10.1186/s12879-023-08466-8 PMC10548573

[28] Ota R, Hata T, Hirata A et al (2023) Risk of infection from glucocorticoid and methotrexate interaction in patients with rheumatoid arthritis using biologics: a retrospective cohort study. Br J Clin Pharmacol 89(7):2168–2178 36755477 10.1111/bcp.15687

[29] Liba T, Alon G, Liron L, Raz G, Elcio M, and Segal O (2025) Epidemiological characterization of uveitis in Japan: a systematic review. Ocul Immunol Inflamm 1–10. 10.1080/09273948.2025.2452193 10.1080/09273948.2025.2452193 39982373

[30] Rice JB, White AG, Scarpati LM, Wan G, Nelson WW (2017) Long-term systemic corticosteroid exposure: a systematic literature review. Clin Ther 39(11):2216–2229 29055500 10.1016/j.clinthera.2017.09.011

[31] Yasir M, Goyal A, Sonthalia S (2024) Corticosteroid adverse effects. StatPearls Publishing, Treasure Island 30285357

[32] Kirupaharan N, Marshall RF, Spangler MD, Armbrust KR, Berkenstock MK (2025) Incidence and Prevalence of uveitis and associated ocular complications in the United States TriNetX Database. Am J Ophthalmol 276;30-39 10.1016/j.ajo.2025.03.032 40157445

[33] Fung AT, Tran T, Lim LL et al (2020) Local delivery of corticosteroids in clinical ophthalmology: a review. Clin Exp Ophthalmol 48(3):366–401 31860766 10.1111/ceo.13702 PMC7187156

[34] Takeuchi M, Kanda T, Kaburaki T et al (2019) Real-world evidence of treatment for relapse of noninfectious uveitis in tertiary centers in Japan: a multicenter study. Medicine 98(9):e14668 30817592 10.1097/MD.0000000000014668 PMC6831171

[35] Chang YC, Kao TE, Chen CL et al (2024) Use of corticosteroids in non-infectious uveitis - expert consensus in Taiwan. Ann Med 56(1):2352019 38747459 10.1080/07853890.2024.2352019 PMC11097703

[36] McHarg M, Young L, Kesav N, Yakin M, Sen HN, Kodati S (2022) Practice patterns regarding regional corticosteroid treatment in noninfectious uveitis: a survey study. J Ophthalmic Inflamm Infect 12(1):3 34982279 10.1186/s12348-021-00281-z PMC8727651

[37] Committee for the Creation of Clinical Guidelines for Uveitis, Japan Society of Ocular Inflammation (2019) Uveitis management guidelines. Nippon Ganka Gakkai Zasshi 123(6):635–696

[38] Jiang Q, Li Z, Tao T, Duan R, Wang X, Su W (2021) TNF-α in uveitis: from bench to clinic. Front Pharmacol 12:740057 34795583 10.3389/fphar.2021.740057 PMC8592912

[39] Pichi F, Smith SD, Goldstein DA et al (2024) The Humira in Ocular inflammations Taper (HOT) study. Am J Ophthalmol 258:87–98 37734639 10.1016/j.ajo.2023.09.012

[40] Almoallim H, Al-Ghamdi Y, Almaghrabi H, Alyasi O (2012) Anti-tumor necrosis factor-α induced systemic lupus erythematosus. Open Rheumatol J 6;315– 319 10.2174/1874312901206010315 PMC3504723 23198006

[41] Li L, Aviña-Zubieta JA, Bernstein CN et al (2023) Risk of multiple sclerosis among users of antitumor necrosis factor α in 4 Canadian provinces: a population-based study. Neurology 100(6):e558–e567 36307225 10.1212/WNL.0000000000201472 PMC9946189

[42] Toussirot É, Aubin F (2016) Paradoxical reactions under TNF-α blocking agents and other biological agents given for chronic immune-mediated diseases: an analytical and comprehensive overview. RMD Open 2(2):e000239 27493788 10.1136/rmdopen-2015-000239 PMC4964220

[43] Hung J-H, Rao NA, Chiu W-C, Sheu S-J (2023) Vitreoretinal surgery in the management of infectious and non-infectious uveitis — a narrative review. Graefes Arch Clin Exp Ophthalmol 261(4):913–923 36220982 10.1007/s00417-022-05862-9

[44] Kim KW, Kusuhara S, Imai H et al (2021) Outcomes of primary 27-gauge vitrectomy for 73 consecutive cases with uveitis-associated vitreoretinal disorders. Front Med (Lausanne) 8:755816 34778318 10.3389/fmed.2021.755816 PMC8578237

[45] Abdulaal MR, Abiad BH, Hamam RN (2015) Uveitis in the aging eye: incidence, patterns, and differential diagnosis. J Ophthalmol 2015(1):509456 26090218 10.1155/2015/509456 PMC4452188

[46] Akinsoji E, Goldhardt R, Galor A (2018) A glimpse into uveitis in the aging eye: pathophysiology, clinical presentation and treatment considerations. Drugs Aging 35(5):399–408 29663152 10.1007/s40266-018-0545-3 PMC5955816

