# Supplementary material for: Real-world treatment patterns for patients with non-infectious uveitis in Japan: a descriptive study using a large-scale claims database (J-CAT study)
Source: J Ophthalmic Inflamm Infect. 2025 Jul 22;15:56. doi: 10.1186/s12348-025-00514-5 (PMC12283504; doi:10.1186/s12348-025-00514-5)
Supplement: Supplementary file 1 — Supplementary Material 1. [file 12348_2025_514_MOESM1_ESM.pdf]

## SUPPLEMENTARY TABLES

**Table S1** Patient diagnosis *ICD-10* codes included[a] from the JMDC database

| ***ICD-10* code** | **Description** |
|---|---|
| C80 | Malignant neoplasm < tumor >, site unspecified |
| D86 | Sarcoidosis |
| E10 | Type 1 < insulin dependent > diabetes mellitus <IDDM> |
| E11 | Type 2 < non-insulin dependent > diabetes mellitus <NIDDM> |
| E14 | Unspecified diabetes mellitus |
| G35 | Multiple sclerosis |
| H15 | Scleral disorder |
| H20 | Iridocyclitis |
| H30 | Choroidal inflammation |
| H31 | Other disorders of the choroid |
| H33 | Retinal detachment and tear |
| H35 | Other retinal disorders |
| H40 | Glaucoma |
| H44 | Eye disorders |
| K50 | Crohn’s disease [localized enteritis] |
| K51 | Ulcerative colitis |
| K90 | Intestinal malabsorption |
| L40 | Psoriasis < psoriasis > |
| L93 | Lupus erythematosus < Lupus erythematosus > |
| M02 | Reactive arthropathy |
| M05 | Seropositive rheumatoid arthritis |
| M06 | Other types of rheumatoid arthritis |
| M08 | Juvenile arthritis |
| M30 | Polyarteritis nodosa and related conditions |
| M31 | Other < destructed > fatal angiopathy |
| M32 | Systemic lupus erythematosus < Lupus erythematosus ><SLE> |
| M35 | Other systemic connective tissue disorders |
| M45 | Ankylosing spondylitis |
| M47 | Spondylosis |
| M94 | Other disorders of cartilage |
| N05 | Unspecified nephritic syndrome |
| N12 | Tubulointerstitial nephritis, not specified as acute or chronic |
| S05 | Injury to eye and orbit |

[a] Patients who had any of these diagnoses at least once between January 1, 2005 and October 31, 2023 were included in the first step of patient selection (Fig. 1).
*ICD-10, International Classification of Diseases, Tenth Revision*.

**Table S2** NIU-related *ICD-10* codes included[a] from the JMDC database

| ***ICD-10* code** | ***ICD-10* name** | **Standard disease code** | **Standard disease name** |
|---|---|---|---|
| C809 | Malignant neoplasm < tumor >; Primary site unspecified | 8845247 | Carcinoma-associated retinopathy |
| D868 | Sarcoidosis in other and multiple sites | 1359003 | Sarcoid uveitis |
| | | 8834028 | Sarcoidosis iridocyclitis |
| | | 1359008 | Ocular sarcoidosis |
| D869 | Sarcoidosis; not otherwise specified | 1359002 | Sarcoidosis |
| E103 | Type 1 < Insulin-dependent > Diabetes mellitus <IDDM>/with ocular complications | 8845053 | Type 1 diabetic iritis |
| E113 | Type 2 < non-insulin dependent > diabetes mellitus <NIDDM>/with ocular complications | 8845082 | Type 2 diabetic iritis |
| E143 | Unspecified diabetes mellitus/with ocular complications | 2504004 | Diabetic iritis |
| H150 | Scleritis | 3790003 | Scleritis |
| | | 3790007 | Posterior scleritis |
| H151 | Episcleritis | 3790006 | Episcleritis |
| H158 | Other disorders of the sclera | 8830862 | Necrotizing scleritis |
| H200 | Acute and subacute iridocyclitis | 8830189 | Subacute anterior uveitis |
| | | 8830193 | Subacute cyclitis |
| | | 3640003 | Subacute iridocyclitis |
| | | 8830182 | Subacute iritis |
| | | 8838292 | Endogenous uveitis |
| | | 3643008 | Hypopyon |
| | | 3643012 | Hypopyon iritis |
| | | 8839042 | Recurrent anterior uveitis |
| | | 3644004 | Recurrent hypopyon |
| | | 8839037 | Recurrent iritis |
| | | 8839038 | Recurrent iridocyclitis |
| | | 8839048 | Recurrent cyclitis |
| | | 3643025 | Acute anterior uveitis |
| | | 8832340 | Acute iritis |
| | | 3640007 | Acute iridocyclitis |
| | | 8832455 | Acute cyclitis |
| | | 3640010 | Serous iritis |
| | | 3640009 | Secondary uveitis |
| | | 8836826 | Secondary iritis |
| | | 3643010 | Secondary iridocyclitis |
| H201 | Chronic iridocyclitis | 8840938 | Rheumatic iritis |
| | | 3641003 | Chronic iridocyclitis |
| H202 | Lenticular iridocyclitis | 8835668 | Lenticular iridocyclitis |
| H208 | Other iridocyclitis | 3642001 | Fuchs heterochromic cyclitis |
| | | 3643027 | Intermediate uveitis |
| | | 8834639 | Hemorrhagic iritis |
| | | 3645002 | Heterochromia iridis |
| | | 8833438 | Heterochromic cyclitis |
| | | 8836076 | Prolonged iritis |
| | | 8837803 | Old iritis |
| | | 3641002 | Old iridocyclitis |
| | | 8838368 | Refractory uveitis |

| *ICD-10* code | *ICD-10* name | Standard disease code | Standard disease name |
|---|---|---|---|
| | | 8833423 | Hypertensive iridocyclitis |
| | | 8839623 | Vogt-Koyanagi disease |
| H209 | Iridocyclitis; not otherwise specified | 3643001 | Uveitis |
| | | 8839834 | Uveitis |
| | | 8831169 | Keratoiritis |
| | | 8833117 | Nodular iritis |
| | | 3643004 | Iritis |
| | | 3643023 | Iridocyclitis |
| | | 8840664 | Cyclitis |
| H300 | Localized inflammation of chorioretinal membrane | 8832661 | Localized choroiditis |
| | | 8832662 | Localized retinitis |
| | | 8832663 | Localized chorioretinitis |
| | | 8833279 | Focal exudative chorioretinitis |
| | | 8838455 | Papilloretinitis |
| H301 | Scattered inflammation of chorioretinal membrane | 8834080 | Disseminated choroiditis |
| | | 8834082 | Disseminated chorioretinitis |
| | | 8834081 | Diffuse retinitis |
| | | 8837479 | Geographic choroiditis |
| | | 3631007 | Diffuse choroiditis |
| | | 8851411 | Shrapnel chorioretinopathy |
| | | 8851449 | Multiple vanishing white spot syndrome |
| H302 | Posterior cyclitis | 8833680 | Posterior cyclitis |
| | | 3631001 | Peripheral uveitis |
| | | 3631002 | Peripheral chorioretinitis |
| | | 8834554 | Peripheral uveitis |
| | | 3631003 | Peripheral choroiditis |
| H308 | Inflammation of other chorioretins | 8839622 | Vogt-Koyanagi-Harada disease |
| H309 | Choroidal inflammation; not otherwise specified | 3632019 | Chorioretinitis |
| | | 3632007 | Choroiditis |
| | | 3632012 | Purulent retinitis |
| | | 8834346 | Neuroretinitis |
| | | 3632025 | Linear retinitis |
| | | 8836821 | Proliferative retinitis |
| | | 3632008 | Retinitis |
| H318 | Other specified disorder of choroid | 8847666 | Punctate choroidal lining |
| H350 | Simple retinopathy and retinal vascular changes | 3621018 | Retinal vasculitis |
| | | 3621040 | Retinal perivasculitis |
| | | 3621023 | Retinal phlebitis |
| | | 3621024 | Retinal perivenous inflammation |
| | | 3621032 | Exudative retinitis |
| H355 | Hereditary retinal dystrophy | 8832346 | Acute posterior multifocal macular pigment epitheliopathy |
| H404 | Glaucoma secondary to eye inflammation | 3656001 | Posner-Schlossman syndrome |
| H441 | Other endophthalmitis | 3601002 | Sympathetic uveitis |
| | | 3601003 | Sympathetic ophthalmia |
| | | 3601004 | Panuveitis |
| | | 3601005 | Lens hypersensitivity endophthalmitis |
| | | 8833447 | Iridocyclitic choroiditis |
| M45- | Ankylosing spondylitis | 8832544 | Ankylosing spondylitis Iridocyclitis |

| ***ICD-10* code** | ***ICD-10* name** | **Standard disease code** | **Standard disease name** |
|---|---|---|---|
| M352 | Behçet's disease | 1361002 | Behçet's disease |
| | | 8846052 | Incomplete Behçet's disease |
| | | 8845881 | Ocular Behçet's disease |

[a] Patients who had a record of any of these NIU-related diseases at least once between January 1, 2005 and October 31, 2023 were included in the second step of patient selection (Fig. 1).
*ICD-10, International Classification of Diseases, Tenth Revision*; NIU, non-infectious uveitis.

**Table S3** Standard ophthalmological examination procedure codes included[a] from the JMDC database

| Standard Procedure Code | Description |
|---|---|
| 160081010 | Full ophthalmoscopy (piece) |
| 160081130 | Full ophthalmoscopy (both) |
| 160203710 | Fundus camera (analog photography) |
| 160203810 | Fundus camera (digital photography) |
| 160081550 | Fundus camera (fluorescence angiography) |
| 160199310 | Fundus camera (autofluorescence) |
| 160199410 | Premium for wide-field fundus photography |
| 160183310 | Three-dimensional imaging analysis of the fundus |
| 160081610 | Slit M (anterior/posterior segment) |
| 160146550 | Vital staining after slit M (anterior and posterior parts) re-examination |
| 160084510 | Slit M (anterior segment) |
| 160084650 | Vital stain after slit M (anterior segment) re-examination |
| 160171110 | Pan-vitreoretinal examination (unilateral) |

[a] Patients who had any of these ophthalmological examinations between October 1, 2016 and October 31, 2023 were included in the third step of patient selection (Fig. 1).

**Table S4** *ICD-10* codes and standard disease codes excluded[a] as non-infectious uveitis from the JMDC database

| *ICD-10* code | *ICD-10* name | Standard disease code | Standard disease name |
|---|---|---|---|
| A185 | Tuberculosis of the eye | 173010 | Tuberculous iridocyclitis |
| | | 8831950 | Eye tuberculosis |
| | | 8833036 | Tuberculous keratoscleritis |
| | | 8833049 | Tuberculous iritis |
| | | 8833078 | Tuberculous uveitis |
| | | 8833080 | Tuberculous chorioretinitis |
| | | 8833081 | Tuberculous retinitis |
| A514 | Other secondary syphilis | 8836649 | Early syphilitic ophthalmopathy |
| | | 8837227 | Secondary syphilitic eye disorder |
| | | 8837229 | Secondary syphilitic iridocyclitis |
| A527 | Other symptomatic late syphilis | 948012 | Syphilitic chorioretinitis |
| | | 8832045 | Ocular syphilis |
| | | 8851318 | Tertiary syphilitic episcleritis |
| A543 | Gonococcal infection in the eye | 8841075 | Gonococcal iridocyclitis |
| B005 | Herpesvirus ophthalmic | 3621046 | Acute retinal necrosis |
| | | 3643026 | Kirisawa uveitis |
| | | 8839979 | Herpetic iritis |
| | | 8839980 | Herpes zoster virus iridocyclitis |
| | | 8839984 | Herpes zoster virus anterior uveitis |
| | | 8839988 | Herpes zoster virus chorioretinitis |
| B023 | Herpes zoster ophthalmic | 8836924 | Herpes zoster scleritis |
| | | 8836926 | Herpes zoster iritis |
| | | 8836927 | Herpes zoster iridocyclitis |
| B580 | Toxoplasma eye disorders | 1309003 | Toxoplasma retinopathy |
| | | 1309004 | Ocular toxoplasmosis |
| | | 8838165 | Toxoplasma chorioretinitis |
| B830 | Visceral larva migrans | 8838156 | Toxocara chorioretinitis |
| H440 | Purulent endophthalmitis | 3600004 | purulent endophthalmitis |
| | | 8834738 | Vitreous abscess |
| | | 8841634 | Endophthalmitis |

[a] Patients who had a record of any of these IU-related diseases at least once after NIU diagnosis were excluded after the fourth step of patient selection (Fig. 1).
*ICD-10, International Classification of Diseases, Tenth Revision*; IU, infectious uveitis.

**Table S5** NIU treatment-related WHO ATC codes included in the study

| Treatment category | | General name | WHO ATC code |
|---|---|---|---|
| Corticosteroid sub-Tenon injections | KENACORT | Triamcinolone acetonide | H02AB08 |
| | MaQaid | Triamcinolone acetonide | S01BA05 |
| | Dexamethasone | Dexamethasone sodium phosphate<br>Dexamethasone metasulfobenzoate sodium<br>Dexamethasone acetate<br>Dexamethasone palmitate | H02AB02 |
| | Betamethasone | Betamethasone acetate/betamethasone sodium phosphate<br>Betamethasone sodium phosphate | H02AB01 |
| Corticosteroid subconjunctival injections | KENACORT | Triamcinolone acetonide | H02AB08 |
| | Dexamethasone | Dexamethasone sodium phosphate<br>Dexamethasone metasulfobenzoate sodium<br>Dexamethasone acetate<br>Dexamethasone palmitate | H02AB02 |
| | Betamethasone | Betamethasone acetate /betamethasone sodium phosphate<br>Betamethasone sodium phosphate | H02AB01 |
| Corticosteroid intravitreal injections | KENACORT | Triamcinolone acetonide | H02AB08 |
| | MaQaid | Triamcinolone acetonide | S01BA05 |
| | Dexamethasone | Dexamethasone sodium phosphate<br>Dexamethasone metasulfobenzoate sodium<br>Dexamethasone acetate<br>Dexamethasone palmitate | H02AB02 |
| | Betamethasone | Betamethasone acetate/betamethasone sodium phosphate<br>Betamethasone sodium phosphate | H02AB01 |
| Corticosteroid drip infusion/intravenous injections | | Methylprednisolone acetate<br>Methylprednisolone sodium succinate | H02AB04 |
| | | Dexamethasone metasulfobenzoate sodium<br>Dexamethasone acetate<br>Dexamethasone sodium phosphate | H02AB02 |
| | | Prednisolone sodium succinate | H02AB06 |
| | | Betamethasone sodium phosphate | H02AB01 |
| | | Hydrocortisone sodium phosphate<br>Hydrocortisone sodium succinate | H02AB09 |
| Oral corticosteroids | | Prednisolone | H02AB06 |
| | | Dexamethasone | H02AB02 |
| | | Betamethasone | H02AB01 |

| Treatment category | | General name | WHO ATC code |
|---|---|---|---|
| | | Triamcinolone | H02AB08 |
| | | Cortisone acetate | H02AB10 |
| | | Methylprednisolone | H02AB04 |
| | | Hydrocortisone | H02AB09 |
| Other corticosteroid injections (pharmacy) | | Dexamethasone sodium phosphate<br>Dexamethasone metasulfobenzoate sodium<br>Dexamethasone acetate<br>Dexamethasone palmitate | H02AB02 |
| | | Betamethasone acetate/betamethasone sodium phosphate<br>Betamethasone sodium phosphate | H02AB01 |
| | | Triamcinolone acetonide | H02AB08 |
| | | Triamcinolone acetonide | S01BA05 |
| Corticosteroid eye drops | | Dexamethasone sodium phosphate<br>Dexamethasone metasulfobenzoate sodium | S03BA01 |
| | | Betamethasone sodium phosphate/fradiomycin sulfate | S03CA06 |
| | | Betamethasone sodium phosphate | S03BA03 |
| | | Fluorometholone | S01BA07 |
| | | Dexamethasone metasulfobenzoate sodium | S01BA01 |
| | | Betamethasone sodium phosphate | S01BA06 |
| | | Hydrocortisone acetate | S01BA02 |
| | | Prednisolone acetate | S01BA04 |
| Immunosuppressants | Immunosuppressants (ophthalmic) | Cyclosporine | S01XA18 |
| | | Tacrolimus hydrate | S01XA |
| | Immunosuppressants (oral) | Methotrexate | L04AX03 |
| | | Mycophenolate mofetil | L04AA06 |
| | | Cyclosporine | L04AD01 |
| | | Azathioprine | L04AX01 |
| | | Cyclophosphamide hydrate | L01AA01 |
| TNF-α inhibitors | Adalimumab | Adalimumab (genetical recombination) | L04AB04 |
| | Infliximab | Infliximab (genetical recombination) | L04AB02 |
| Other (JAK inhibitors, PDE4 inhibitors, colchicine) | JAK inhibitors | Ritresitinib tosilate | L04AF08 |
| | | Tofacitinib citrate | L04AA29 |
| | | Ruxolitinib phosphate | L01EJ01 |
| | | Baricitinib | L04AA37 |
| | | Peficitinib hydrobromide | L04AA49 |
| | | Upadacitinib hydrate | L04AA44 |
| | | Filgotinib maleate | L04AA45 |
| | | Deucravacitinib | L04AA56 |
| | PDE4 inhibitor | Apremilast | L04AA32 |
| | Colchicine | Colchicine | M04AC01 |

ATC, Anatomical Therapeutic Chemical Classification System; NIU, non-infectious uveitis; WHO, World Health Organization.

**Table S6** *ICD-10* codes for glaucoma, UME, and ERM included in the study

| ***ICD-10* code** | ***ICD-10* name** | **Standard disease code** | **Standard disease name** |
|---|---|---|---|
| E720 | Amino acid transfer disorder | 8841216 | Lowe syndrome glaucoma |
| E859 | Amyloidosis < amyloidosis >, unspecified | 8849864 | Amyloid glaucoma |
| H353 | Degeneration of macula and posterior pole | 8840643 | Preretinal membrane |
| H358 | Other specified retinal disorder | 3625009 | Macular edema |
| | | 8838768 | Cystoid macular edema |
| H400 | Suspected glaucoma | 8845275 | Primary angle closure |
| | | 3650005 | Ocular hypertension |
| | | 8850670 | Anterior visual field glaucoma |
| H401 | Primary open-angle glaucoma | 8831041 | Open-angle glaucoma |
| | | 3651010 | Primary open-angle glaucoma |
| | | 3651004 | Pigmentary glaucoma |
| | | 3651011 | Normal tension glaucoma |
| | | 3651007 | Chronic open-angle glaucoma |
| H402 | Primary angle-closure glaucoma | 3652013 | Malignant glaucoma |
| | | 8832444 | Acute angle-closure glaucoma |
| | | 3652015 | Acute glaucoma attack |
| | | 3652006 | Primary angle-closure glaucoma |
| | | 8840397 | Chronic angle-closure glaucoma |
| H404 | Glaucoma secondary to eye inflammation | 8832290 | Acute inflammatory glaucoma |
| H405 | Glaucoma secondary to other eye disorders | 8831440 | Hypersecretory glaucoma |
| | | 3651012 | Neovascular glaucoma |
| | | 3659001 | Hemorrhagic glaucoma |
| | | 3655002 | Lenticular glaucoma |
| | | 3655004 | Phacolytic glaucoma |
| | | 3659002 | Secondary glaucoma |
| | | 3655003 | Aphakic glaucoma |
| | | 8840785 | Hemolytic glaucoma |
| | | 8849220 | Exfoliation glaucoma |
| H406 | Drug-induced secondary glaucoma | 3653002 | Steroid glaucoma |
| | | 8840739 | Drug-induced glaucoma |
| H408 | Other glaucoma | 8830432 | Iatrogenic glaucoma |
| | | 8833889 | Mixed glaucoma |
| H409 | Glaucoma, unspecified | 3659005 | Primary glaucoma |
| | | 3659003 | Glaucoma |
| | | 8850699 | Glaucomatous optic atrophy |
| | | 8850700 | Glaucomatous optic neuropathy |
| | | 8846067 | Glaucomatous cupping of papilla |

ERM, epiretinal membrane; *ICD-10, International Classification of Diseases, Tenth Revision*; UME, uveitic macular edema.

**Table S7** *ICD-10* codes for disease comorbidities included in the study

| ***ICD-10* code** | ***ICD-10* name** | **Disease category** |
|---|---|---|
| D869 | Sarcoidosis; Not otherwise specified | Sarcoidosis |
| E14- | Unspecified diabetes mellitus | Unspecified diabetes mellitus |
| E140 | Unspecified diabetes mellitus/with coma | |
| E141 | Unspecified diabetes mellitus/with ketoacidosis | |
| E142 | Unspecified diabetes mellitus/with renal complications | |
| E143 | Unspecified diabetes mellitus/with ocular complications | |
| E144 | Unspecified diabetes mellitus/with neurological (neurological) complications | |
| E145 | Unspecified diabetes mellitus/with peripheral circulatory complications | |
| E146 | Unspecified diabetes mellitus/with other specified complication | |
| E149 | Unspecified diabetes mellitus/Uncomplicated | |
| G35- | Multiple sclerosis | Multiple sclerosis |
| K500 | Crohn’s disease of the small intestine | Crohn’s disease [localized enteritis] |
| K501 | Crohn’s disease of the large intestine | |
| K508 | Other Crohn’s disease | |
| K509 | Crohn’s disease, unspecified | |
| K510 | Ulcerative (chronic) pancolitis | Ulcerative colitis |
| K512 | Ulcerative (chronic) proctitis | |
| K513 | Ulcerative (chronic) proctosigmoiditis | |
| K515 | Left-sided colitis | |
| K518 | Other ulcerative colitis | |
| K519 | Colitis ulcerative, unspecified | |
| K900 | Pediatric steatorrhea | Intestinal malabsorption |
| K902 | Blind loop < blind loop > syndrome, not elsewhere classified | |
| K904 | Malabsorption due to intolerance, not elsewhere classified | |
| K908 | Other intestinal malabsorption | |
| K909 | Intestinal malabsorption, unspecified | |
| L400 | Psoriasis vulgaris < psoriasis > | Psoriasis < psoriasis > |
| L401 | Generalized pustular psoriasis < psoriasis > | |
| L402 | Retained < continuous > acrodermatitis | |
| L403 | Palmoplantar < palmar-plantar > pustulosis | |
| L404 | Guttate psoriasis < psoriasis > | |
| L405 | Psoriasis arthropathica < psoriasis > | |
| L408 | Other forms of psoriasis < psoriasis > | |
| L409 | Psoriasis, unspecified | |
| L930 | Discoid lupus erythematosus < lupus erythematosus ><DLE> | Lupus erythematosus < Lupus erythematosus > |
| L931 | Subacute cutaneous lupus erythematosus < lupus erythematosus > | |
| L932 | Other localized lupus erythematosus < lupus erythematosus > | |
| M022 | Post-vaccination arthropathy | Reactive arthropathy |
| M023 | Reiter’s disease | |
| M029 | Reactive arthropathy, unspecified | |
| M050 | Felty’s syndrome | Seropositive rheumatoid arthritis |

| *ICD-10* code | *ICD-10* name | Disease category |
|---|---|---|
| M051 | Rheumatic lung disease <M051> | |
| M052 | Rheumatoid vasculitis | |
| M053 | Rheumatoid Arthritis with Concurrent Diseases of Other Organs and Organ Systems | |
| M058 | Other seropositive rheumatoid arthritis | |
| M059 | Seropositive rheumatoid arthritis, unspecified | |
| M060 | Seronegative rheumatoid arthritis | Other types of rheumatoid arthritis |
| M061 | adult-onset Still’s disease | |
| M062 | Rheumatic bursitis | |
| M063 | Rheumatoid subcutaneous nodule | |
| M064 | Inflammatory polyarthropathy | |
| M068 | Other specified rheumatoid arthritis | |
| M069 | Rheumatoid arthritis, unspecified | |
| M080 | Juvenile rheumatoid arthritis | Juvenile arthritis |
| M081 | Juvenile ankylosing spondylitis | |
| M082 | Juvenile arthritis with systemic onset [Still’s disease] | |
| M083 | Juvenile polyarthritis (seronegative) | |
| M084 | Juvenile oligoarticular arthritis | |
| M088 | Other juvenile arthritis | |
| M089 | Juvenile arthritis, unspecified | |
| M300 | Polyarteritis nodosa | Polyarteritis nodosa and related conditions |
| M301 | Multiple (multiple) arteritis with pulmonary involvement [Churg-Strauss syndrome] | |
| M302 | Juvenile polyarteritis (polyarteritis) | |
| M303 | Mucocutaneous lymph node syndrome [Kawasaki’s disease] | |
| M308 | Other conditions related to polyarteritis nodosa | |
| M310 | Hypersensitivity vasculitis | Other < destructed > fatal angiopathy |
| M311 | Thrombotic microangiopathy | |
| M312 | Bleeding rhinitis <Idiopathic nasal emphysema> | |
| M313 | Wege granuloma | |
| M314 | Aortic arch syndrome [Takayasu disease] | |
| M316 | Other giant cell (sexual) arteritis | |
| M317 | Microscopic multifocal vasculitis | |
| M318 | Other specific < destruction > death vascular disorder | |
| M319 | Vascular death, unspecified | |
| M320 | Drug-induced systemic lupus erythematosus < Lupus erythematous ><SLE> | Systemic lupus erythematosus < Lupus erythematosus ><SLE> |
| M321 | Systemic Lupus Erythematosus with Concurrent Organ or Organ System < Lupus Erythematosus ><SLE> | |
| M329 | Systemic lupus erythematosus < lupus erythematosus ><SLE>, unspecified | |
| M352 | Behçet’s disease | Behçet’s disease |
| M470 | Anterior spinal artery and vertebral artery compression syndrome | Spondylosis |
| M471 | Other spondylopathies with myelopathy | |
| M472 | Other spondylosis with radiculopathy | |
| M478 | Other spondylosis | |
| M479 | Spinopathy, unspecified | |
| M940 | Costochondral bone union syndrome [Tietze’s disease] | Other disorders of cartilage |
| M941 | Relapsing polychondritis | |

| *ICD-10* code | *ICD-10* name | Disease category |
|---|---|---|
| M942 | Chondromalacia | |
| M943 | Chondrolysis | |
| M948 | Other specified disorder of cartilage | |
| N050 | Unspecified nephritic syndrome/minimal glomerular changes | Unspecified nephritic syndrome |
| N051 | Unspecified nephritic syndrome/focal and segmental glomerular lesions | |
| N052 | Unspecified nephritic syndrome/diffuse membranous glomerulonephritis | |
| N053 | Unspecified nephritic syndrome/diffuse mesangial proliferative glomerulonephritis | |
| N054 | Unspecified nephritic syndrome/diffuse intraductal proliferative glomerulonephritis | |
| N055 | Unspecified nephritic syndrome/diffuse mesangial capillary glomerulonephritis | |
| N056 | Unspecified Nephritic Syndrome/Dense Deposit Disease | |
| N057 | Unspecified nephritic syndrome/diffuse crescentic (forming) glomerulonephritis | |
| N058 | Unspecified nephritic syndrome/other | |
| N059 | Unspecified nephritic syndrome/Unspecified | |
| N12- | Tubulointerstitial nephritis, not specified as acute or chronic | Tubulointerstitial nephritis |

*ICD, International Classification of Diseases, Tenth Revision*.

**Table S8** Patient demographics and clinical characteristics in patients with moderate-to-severe NIU who were initially treated with local/systemic corticosteroids (except eye drops)

| | Corticosteroids | | | | | |
|---|---|---|---|---|---|---|
| **Characteristic, %** | **Oral *n* = 6,588** | **ST inj *n* = 1,320** | **SCJ inj *n* = 897** | **IV inj *n* = 708** | **IVT inj *n* = 69** | **Other local inj *n* = 94** |
| Age group | | | | | | |
| 0–19 years | 8.58 | 2.80 | 4.01 | 7.91 | 0 | 3.19 |
| 20–39 years | 21.75 | 16.29 | 24.64 | 21.33 | 4.35 | 12.77 |
| 40–59 years | 52.90 | 52.50 | 53.51 | 53.10 | 59.40 | 46.80 |
| ≥ 60 years | 16.80 | 28.40 | 17.84 | 17.70 | 36.20 | 37.20 |
| Sex, male | 48.30 | 50.80 | 56.86 | 49.70 | 53.60 | 48.90 |
| NIU diagnosis[a] | | | | | | |
| Sarcoidosis | 3.48 | 4.39 | 2.34 | 2.68 | 0 | 5.32 |
| Behçet’s disease | 2.34 | 0.68 | 1.11 | 2.26 | 1.45 | 0 |
| Posner-Schlossman syndrome | 0.84 | 0.38 | 0.56 | 1.55 | 0 | 1.06 |
| Vogt-Koyanagi-Harada disease | 6.00 | 2.35 | 0.56 | 14.97 | 0 | 7.45 |
| Acute anterior uveitis | 0.67 | 0.99 | 5.69 | 0.14 | 0 | 1.06 |
| Diabetic iritis | 0.05 | 0.38 | 0.56 | 0 | 1.45 | 0 |
| Multiple Evanescent White Dot Syndrome | 0.05 | 0.08 | 0 | 0 | 0 | 0 |
| Rheumatoid arthritis-associated uveitis | 0 | 0.15 | 0.11 | 0 | 0 | 0 |
| Fuchs iridocyclitis | 0 | 0 | 0 | 0 | 0 | 0 |
| Diagnosis with systemic disorder associated with NIU[b] | 10.69 | 11.97 | 9.36 | 9.18 | 21.74 | 12.77 |
| Treatment for systemic disorders related to NIU[b] | 18.62 | 5.00 | 4.91 | 14.55 | 1.45 | 9.57 |
| Uveitic macular edema[b] | 0.71 | 5.76 | 1.00 | 0.85 | 8.70 | 4.26 |
| Epiretinal membrane[b] | 1.29 | 5.53 | 2.12 | 0.85 | 7.25 | 3.19 |

[a] Some patients were diagnosed with multiple conditions. [b] ≤ 6 months before index date. Inj, injection; IV, intravenous; IVT, intravitreal; NIU, non-infectious uveitis; SCJ, subconjunctival; ST, sub-Tenon’s.

**Table S9** Patient demographics and clinical characteristics in patients with moderate-to-severe NIU who were initially treated with immunosuppressants or biologics/other

| | Immunosuppressants | | Biologics and other[a] | | | | |
|---|---|---|---|---|---|---|---|
| | Oral | Local[b] | PDE4 inh | JAK inh | Colchicine | Infliximab | Adalimumab |
| Characteristic, % | *n* = 672 | *n* = 73 | *n* = 51 | *n* = 34 | *n* = 421 | *n* = 58 | *n* = 82 |
| Age group | | | | | | | |
| 0–19 years | 6.40 | 34.25 | 0 | 5.88 | 4.99 | 10.34 | 17.07 |
| 20–39 years | 13.39 | 23.29 | 21.57 | 11.76 | 29.69 | 29.31 | 21.95 |
| 40–59 years | 55.95 | 31.51 | 66.67 | 47.06 | 53.44 | 50.00 | 47.56 |
| ≥ 60 years | 24.26 | 10.96 | 11.76 | 35.29 | 11.88 | 10.34 | 13.41 |
| Sex, male | 32.29 | 63.01 | 49.02 | 47.06 | 56.53 | 62.07 | 50.00 |
| NIU diagnosis[c] | | | | | | | |
| Sarcoidosis | 3.42 | 0 | 0 | 2.94 | 1.90 | 3.45 | 3.66 |
| Behçet’s disease | 6.99 | 1.37 | 27.45 | 2.94 | 49.17 | 22.41 | 20.73 |
| Posner-Schlossman syndrome | 0.74 | 0 | 0 | 0 | 0.71 | 1.72 | 0 |
| Vogt-Koyanagi-Harada disease | 1.34 | 0 | 0 | 0 | 0.48 | 0 | 0 |
| Acute anterior uveitis | 0.60 | 0 | 0 | 0 | 0.48 | 1.72 | 1.22 |
| Diabetic iritis | 0 | 0 | 0 | 0 | 0.24 | 0 | 0 |
| Multiple Evanescent White Dot Syndrome | 0 | 0 | 0 | 0 | 0 | 0 | 0 |
| Rheumatoid arthritis-associated uveitis | 0.15 | 0 | 0 | 0 | 0.24 | 0 | 0 |
| Fuchs iridocyclitis | 0 | 0 | 0 | 0 | 0 | 0 | 0 |
| Diagnosis with systemic disorder associated with NIU[d] | 27.83 | 8.22 | 29.41 | 20.59 | 28.03 | 24.14 | 29.27 |
| Treatment for systemic disorders related to NIU[d] | 73.81 | 2.74 | 58.82 | 52.94 | 46.32 | 82.76 | 76.83 |
| Uveitic macular edema[d] | 1.34 | 1.37 | 1.96 | 0 | 0.48 | 1.72 | 0 |
| Epiretinal membrane[d] | 1.64 | 2.74 | 1.96 | 0 | 0.24 | 1.72 | 0 |

[a] Infliximab and adalimumab are TNF-α inhibitors. [b] Eye drops. [c] Some patients were diagnosed with multiple conditions. [d] ≤ 6 months before index date. Inh, inhibitor; JAK, Janus kinase; NIU, non-infectious uveitis; PDE4, phosphodiesterase-4; TNF-α, tumor necrosis factor-alpha.

**Table S10** Patient demographics and clinical characteristics in patients with moderate-to-severe NIU who had been treated with local/systemic corticosteroids (except eye drops) before the last encounter

| Characteristic, % | Corticosteroids | | | | | |
|---|---|---|---|---|---|---|
| | Oral *n* = 7,473 | ST inj *n* = 1,636 | SCJ inj *n* = 1,046 | IV inj *n* = 1,117 | IVT inj *n* = 109 | Other local inj *n* = 123 |
| Age group | | | | | | |
| 0–19 years | 8.31 | 2.63 | 4.21 | 7.25 | 0.92 | 4.88 |
| 20–39 years | 21.77 | 17.42 | 24.76 | 19.70 | 6.42 | 15.45 |
| 40–59 years | 53.10 | 53.60 | 53.40 | 54.30 | 53.20 | 44.70 |
| ≥ 60 years | 16.83 | 26.34 | 17.59 | 18.80 | 39.45 | 34.96 |
| Sex, male | 48.30 | 51.0 | 55.40 | 49.70 | 55.00 | 48.00 |
| NIU diagnosis[a] | | | | | | |
| Sarcoidosis | 3.52 | 4.40 | 2.39 | 2.69 | 0 | 4.88 |
| Behçet's disease | 2.96 | 1.16 | 1.15 | 3.94 | 1.83 | 0 |
| Posner-Schlossman syndrome | 0.87 | 0.37 | 0.48 | 1.25 | 0 | 0.81 |
| Vogt-Koyanagi-Harada disease | 6.97 | 5.50 | 1.63 | 11.73 | 0 | 8.13 |
| Acute anterior uveitis | 0.87 | 1.53 | 5.64 | 0.18 | 0 | 0.81 |
| Diabetic iritis | 0.05 | 0.43 | 0.48 | 0 | 1.83 | 0 |
| Multiple Evanescent White Dot Syndrome | 0.04 | 0.06 | 0 | 0 | 0 | 0 |
| Rheumatoid arthritis-associated uveitis | 0 | 0.12 | 0.10 | 0 | 0 | 0 |
| Fuchs iridocyclitis | 0 | 0 | 0 | 0 | 0 | 0 |
| Diagnosis with systemic disorder associated with NIU[b] | 10.95 | 12.16 | 9.46 | 12.71 | 20.18 | 13.82 |
| Treatment for systemic disorders related to NIU[b] | 19.14 | 7.15 | 6.12 | 22.11 | 4.59 | 10.57 |
| Uveitic macular edema[b] | 0.88 | 5.07 | 0.96 | 1.25 | 9.17 | 4.07 |
| Epiretinal membrane[b] | 1.33 | 4.71 | 1.91 | 1.25 | 7.34 | 2.44 |

[a] Some patients were diagnosed with multiple conditions. [b] ≤ 6 months before index date. Inj, injection; IV, intravenous; IVT, intravitreal; NIU, non-infectious uveitis; SCJ, subconjunctival; ST, sub-Tenon's.

**Table S11** Patient demographics and clinical characteristics in patients with moderate-to-severe NIU who had been treated with immunosuppressants or biologics/other before the last encounter

| | Immunosuppressants | | Biologics and other[a] | | | | |
|---|---|---|---|---|---|---|---|
| **Characteristic, %** | **Oral** ***n*** **= 1,104** | **Local[b]** ***n*** **= 95** | **PDE4 inh** ***n*** **= 88** | **JAK inh** ***n*** **= 91** | **Colchicine** ***n*** **= 553** | **Infliximab** ***n*** **= 108** | **Adalimumab** ***n*** **= 220** |
| Age group | | | | | | | |
| 0–19 years | 7.43 | 28.42 | 3.41 | 10.99 | 5.42 | 9.26 | 18.64 |
| 20–39 years | 17.12 | 22.11 | 25.00 | 14.29 | 32.01 | 29.63 | 25.91 |
| 40–59 years | 54.90 | 37.90 | 60.20 | 53.80 | 51.60 | 51.90 | 45.90 |
| ≥ 60 years | 20.56 | 11.58 | 11.36 | 20.88 | 11.01 | 9.26 | 9.55 |
| Sex, male | 38.40 | 60.00 | 43.20 | 49.45 | 57.60 | 60.20 | 51.80 |
| NIU diagnosis[c] | | | | | | | |
| Sarcoidosis | 4.26 | 0 | 0 | 4.40 | 1.99 | 4.63 | 4.09 |
| Behçet's disease | 6.79 | 1.05 | 35.23 | 7.69 | 44.77 | 24.07 | 16.36 |
| Posner-Schlossman syndrome | 0.73 | 0 | 0 | 1.10 | 0.54 | 0.93 | 0 |
| Vogt-Koyanagi-Harada disease | 6.43 | 2.11 | 1.14 | 0 | 0.90 | 0 | 9.09 |
| Acute anterior uveitis | 0.82 | 0 | 1.14 | 0 | 0.54 | 1.85 | 1.36 |
| Diabetic iritis | 0 | 0 | 0 | 0 | 0.18 | 0 | 0 |
| Multiple Evanescent White Dot Syndrome | 0 | 0 | 0 | 0 | 0 | 0 | 0 |
| Rheumatoid arthritis-associated uveitis | 0.09 | 0 | 0 | 0 | 0.18 | 0 | 0 |
| Fuchs iridocyclitis | 0 | 0 | 0 | 0 | 0 | 0 | 0 |
| Diagnosis with systemic disorder associated with NIU[d] | 27.73 | 6.32 | 31.82 | 26.37 | 24.37 | 25.93 | 23.82 |
| Treatment for systemic disorders related to NIU[d] | 57.79 | 10.53 | 54.55 | 58.24 | 43.22 | 63.89 | 47.73 |
| Uveitic macular edema[d] | 1.45 | 1.05 | 1.14 | 0 | 0.90 | 0.93 | 0 |
| Epiretinal membrane[d] | 1.36 | 2.11 | 3.41 | 0 | 0.72 | 0.93 | 0.46 |

[a] Infliximab and adalimumab are TNF-α inhibitors. [b] Eye drops. [c] Some patients were diagnosed with multiple conditions. [d] ≤ 6 months before index date. Inh, inhibitor; JAK, Janus kinase; NIU, non-infectious uveitis; PDE4, phosphodiesterase-4; TNF-α, tumor necrosis factor-alpha.

## SUPPLEMENTARY FIGURES

**Fig. S1** Treatment patterns in patients with moderate-to-severe NIU receiving TNF-α inhibitors **(A)** adalimumab and **(B)** infliximab. Only nodes of $n$ = ≥10 are shown.

ADA, adalimumab; col, colchicine; CS, corticosteroid; IFX, infliximab; IS, immunosuppressant; IV, intravenous; M0; no previous treatment; NIU, non-infectious uveitis; TNF-α, tumor necrosis factor-alpha.

## Figure S1A

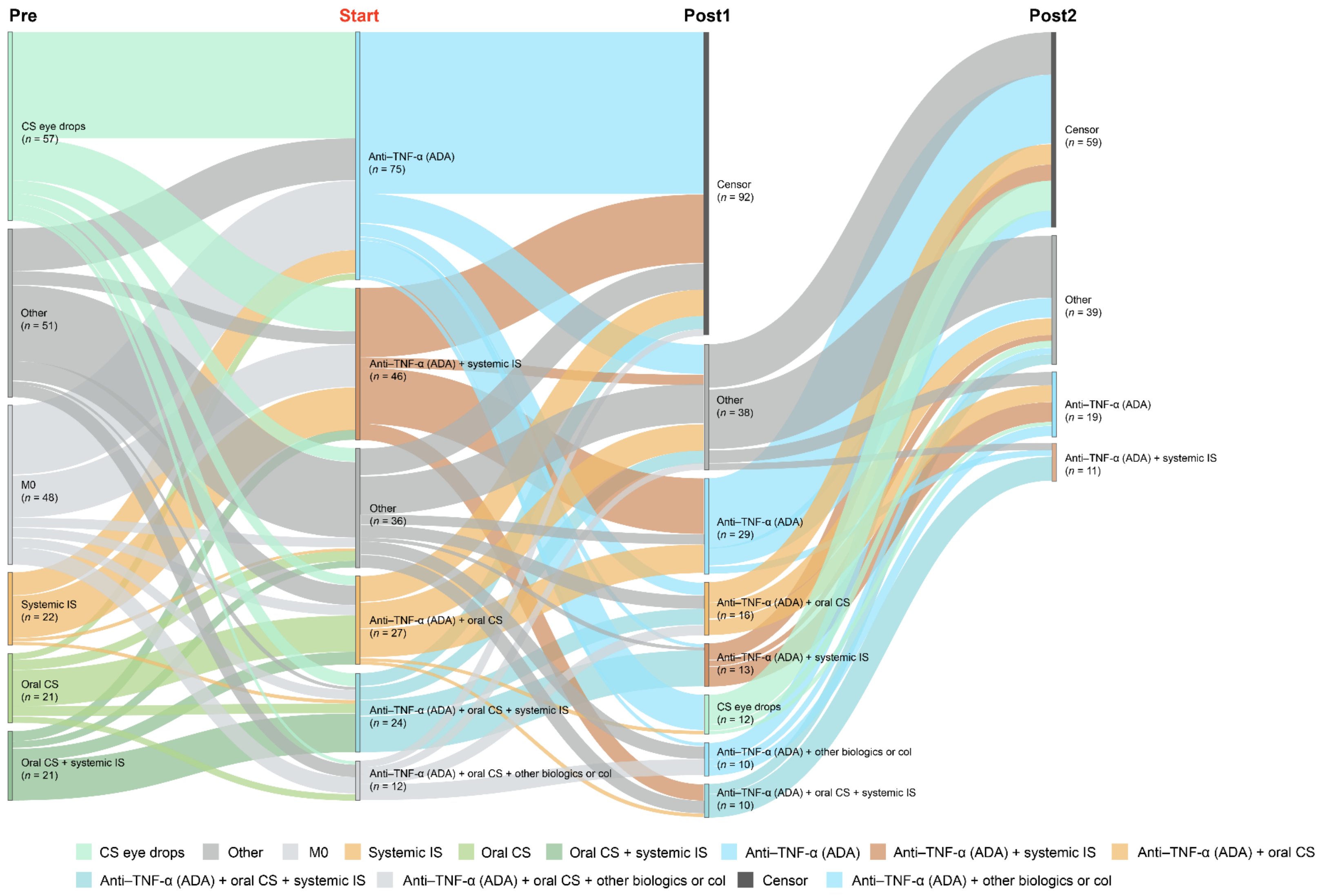
Pre
Start
Post1
Post2
CS eye drops (n = 57)
Other (n = 51)
M0 (n = 48)
Systemic IS (n = 22)
Oral CS (n = 21)
Oral CS + systemic IS (n = 21)
Anti–TNF-α (ADA) (n = 75)
Anti–TNF-α (ADA) + systemic IS (n = 46)
Other (n = 36)
Anti–TNF-α (ADA) + oral CS (n = 27)
Anti–TNF-α (ADA) + oral CS + systemic IS (n = 24)
Anti–TNF-α (ADA) + oral CS + other biologics or col (n = 12)
Censor (n = 92)
Other (n = 38)
Anti–TNF-α (ADA) (n = 29)
Anti–TNF-α (ADA) + oral CS (n = 16)
Anti–TNF-α (ADA) + systemic IS (n = 13)
CS eye drops (n = 12)
Anti–TNF-α (ADA) + other biologics or col (n = 10)
Anti–TNF-α (ADA) + oral CS + systemic IS (n = 10)
Censor (n = 59)
Other (n = 39)
Anti–TNF-α (ADA) (n = 19)
Anti–TNF-α (ADA) + systemic IS (n = 11)
CS eye drops
Other
M0
Systemic IS
Oral CS
Oral CS + systemic IS
Anti–TNF-α (ADA)
Anti–TNF-α (ADA) + systemic IS
Anti–TNF-α (ADA) + oral CS
Anti–TNF-α (ADA) + oral CS + systemic IS
Anti–TNF-α (ADA) + oral CS + other biologics or col
Censor
Anti–TNF-α (ADA) + other biologics or col

## Figure S1B

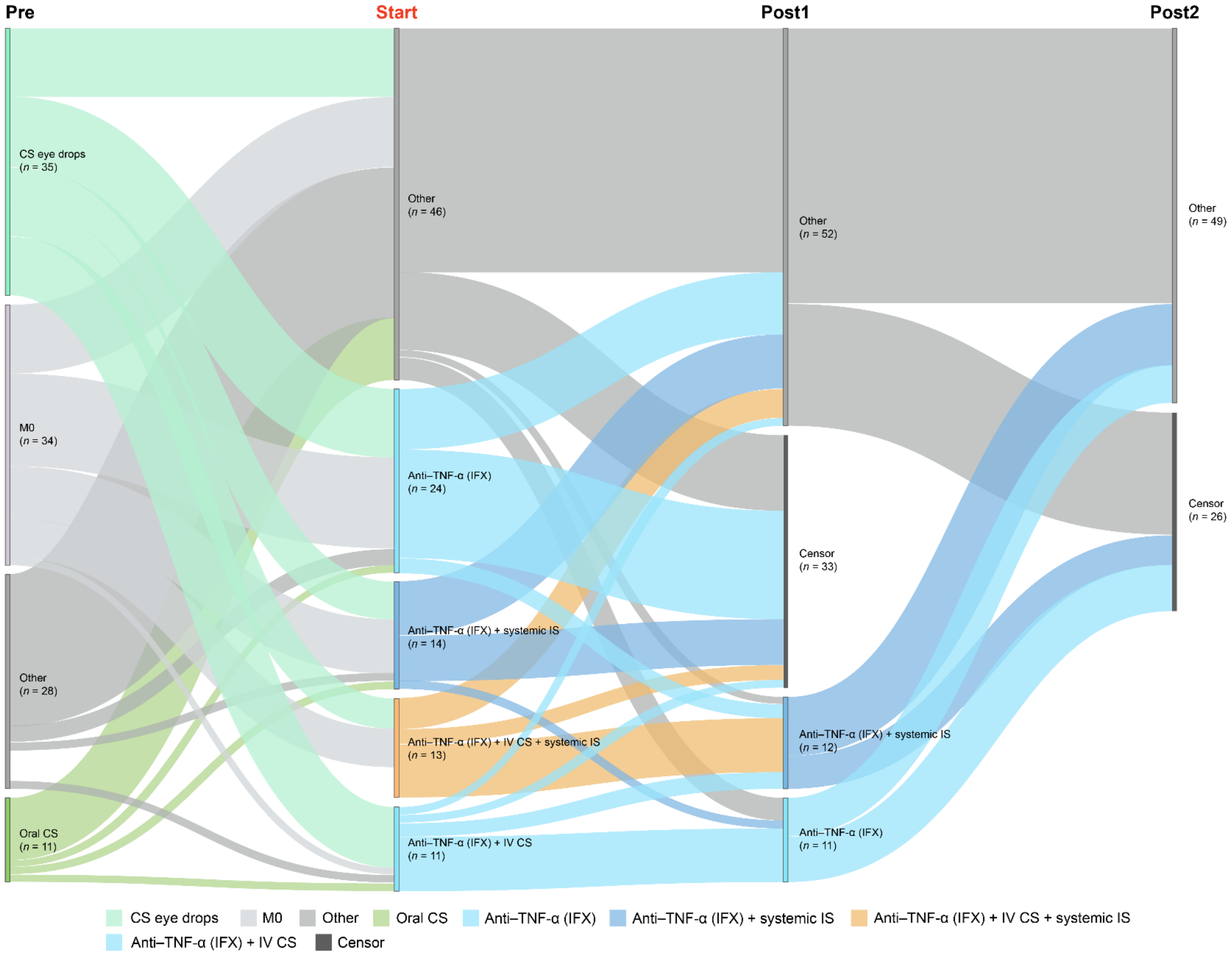

Pre
Start
Post1
Post2
CS eye drops (n = 35)
M0 (n = 34)
Other (n = 28)
Oral CS (n = 11)
Other (n = 46)
Anti–TNF-α (IFX) (n = 24)
Anti–TNF-α (IFX) + systemic IS (n = 14)
Anti–TNF-α (IFX) + IV CS + systemic IS (n = 13)
Anti–TNF-α (IFX) + IV CS (n = 11)
Other (n = 52)
Censor (n = 33)
Anti–TNF-α (IFX) + systemic IS (n = 12)
Anti–TNF-α (IFX) (n = 11)
Other (n = 49)
Censor (n = 26)
CS eye drops
M0
Other
Oral CS
Anti–TNF-α (IFX)
Anti–TNF-α (IFX) + systemic IS
Anti–TNF-α (IFX) + IV CS + systemic IS
Anti–TNF-α (IFX) + IV CS
Censor
